# Smart responsive *in situ* hydrogel systems applied in bone tissue engineering

**DOI:** 10.3389/fbioe.2024.1389733

**Published:** 2024-05-28

**Authors:** Shunli Wu, Tingting Gai, Jie Chen, Xiguang Chen, Weikai Chen

**Affiliations:** ^1^ College of Marine Life Sciences, Ocean University of China, Qingdao, China; ^2^ Hangzhou Singclean Medical Products Co., Ltd, Hangzhou, China; ^3^ School of Medicine, Shanghai University, Shanghai, China; ^4^ Jiaxing Vocational Technical College, Department of Student Affairs, Jiaxing, China; ^5^ College of Marine Life Science, Ocean University of China, Qingdao, China; ^6^ Laoshan Laboratory, Qingdao, China; ^7^ Department of Orthopedics, The Second Affiliated Hospital and Yuying Children’s Hospital of Wenzhou Medical University, Wenzhou, China

**Keywords:** exogenous stimulus, endogenous stimulus, *in situ* hydrogels, smart hydrogels, bone tissue engineering

## Abstract

The repair of irregular bone tissue suffers severe clinical problems due to the scarcity of an appropriate therapeutic carrier that can match dynamic and complex bone damage. Fortunately, stimuli-responsive *in situ* hydrogel systems that are triggered by a special microenvironment could be an ideal method of regenerating bone tissue because of the injectability, *in situ* gelatin, and spatiotemporally tunable drug release. Herein, we introduce the two main stimulus-response approaches, exogenous and endogenous, to forming *in situ* hydrogels in bone tissue engineering. First, we summarize specific and distinct responses to an extensive range of external stimuli (e.g., ultraviolet, near-infrared, ultrasound, etc.) to form *in situ* hydrogels created from biocompatible materials modified by various functional groups or hybrid functional nanoparticles. Furthermore, “smart” hydrogels, which respond to endogenous physiological or environmental stimuli (e.g., temperature, pH, enzyme, etc.), can achieve *in situ* gelation by one injection *in vivo* without additional intervention. Moreover, the mild chemistry response-mediated *in situ* hydrogel systems also offer fascinating prospects in bone tissue engineering, such as a Diels–Alder, Michael addition, thiol-Michael addition, and Schiff reactions, etc. The recent developments and challenges of various smart *in situ* hydrogels and their application to drug administration and bone tissue engineering are discussed in this review. It is anticipated that advanced strategies and innovative ideas of *in situ* hydrogels will be exploited in the clinical field and increase the quality of life for patients with bone damage.

## 1 Introduction

As the load-bearing structure of the human body, the bone skeleton plays crucial functions, such as constituting a motion system and protecting nerves and organs (Harada and Rodan, 2003; Wu and Chang, 2014). However, the bone tissue easily suffers damage due to crashing, falling, infection, and age (Stolzing et al., 2008). Although bone tissue possesses a strong self-repair capacity, it fails to self-heal when the defect exceeds a certain boundary. Therefore, the reconstruction of bone tissue is an extremely significant challenge for clinical surgeons. Several approaches have been exploited for treating bone damage, relying on various therapies (e.g., autografts, xenografts, and allografts) ((Harada and Rodan, 2003; Wu and Chang, 2014). However, these approaches still have challenges, such as donor site breakage, large segmental bone loss, morphology structure mismatch, etc. (Stolzing et al., 2008). To deal with those challenges, many therapeutic strategies using biomaterial-based systems (e.g., bone tissue engineering, bone organoids, etc.) have been exploited and utilized *in vivo* and *in vitro* and have demonstrated a satisfactory therapeutic effect (Liu S. et al., 2023).

The extracellular matrix (ECM) plays a vital role in signaling transfer and the exchange of nutrition and oxygen with emergent tissue (Shang et al., 2021). When designing novel biomaterial systems, it is necessary to match the properties of ECM to mimic the microenvironment of cell proliferative differentiation and improve bone tissue regeneration (Li J. et al., 2023). Hydrogels, an excellent biocompatible replacement material for the ECM, are considered to be the most promising applicants in bone tissue engineering because of their unique 3D-network structure, water-swellable, and adjustable physical and chemical properties (Cui et al., 2019). The hydrogel can also function as a carrier and depot for the delivery of drugs, bioactive peptides, and filling agents to adjust the tissue regeneration processes and boost the development of novel natural tissue (Zheng et al., 2023; Goh et al., 2024; Kalairaj et al., 2024; Park et al., 2024). Various hydrogel-based biomaterials have been extensively utilized in bone tissue regeneration, such as chitosan, gelatin, hyaluronic acid (HA), etc. (Neves et al., 2020; Zhuang and Cui, 2021). In addition, hydrogels have the potential to enhance the proliferation, adhesion, and differentiation properties of bone marrow mesenchymal stromal cells (BMSCs) during bone tissue regeneration (Chaudhuri et al., 2016). Thus, hydrogels are attractive biomaterials in bone tissue regeneration.

The synthesis and application of hydrogels have developed rapidly based on advanced technology, which paves a significant way for bone tissue regeneration (Choi et al., 2021; Yan et al., 2021; Zhang et al., 2022). Smart hydrogels are becoming more attractive in the biomaterial fields for promising applications (Balakrishnan and Banerjee, 2011; Chen et al., 2023). These strategies present several novel possibilities to overcome the shortcomings of traditional materials in bone tissue regeneration, including weak bioactivity and lack of spatial-temporal regulation (Xue et al., 2022a; Kurian et al., 2022). Injectable *in situ* hydrogels are usually modified to mimic the cell environment in terms of having similar properties to the ECM; they can match any defect shape (Liu et al., 2017; Hernández-González et al., 2020). Furthermore, the *in situ* hydrogel system used as a filler and carrier can be loaded with growth factors or bioactive molecules to enhance bone tissue regeneration and avoid surgical intervention in a nidus (Saravanan et al., 2019).

ISmart *in situ* hydrogels can be transformed from fluid to gel under external stimuli and can be used to fill and treat various irregularly shaped bone tissue defects. However, injectable hydrogels (which were in a gel state prior to injection) do not achieve this function. Although both can be stimulus-responsive, the stimulus response described in this article is mainly to realize the transformation of the gel precursor from a liquid state to a gel state *in vivo*. Traditional transplant hydrogels are manufactured before implantation and cannot be dynamically adjusted in an externally controlled and user-defined manner. Compared with traditional prefabricated surgical implant scaffolds, the most significant properties of these hydrogels is that they can be easily injected into the default site *in situ* and form a solid hydrogel, which helps to resist geometric deformation (Fan and Wang, 2017). However, the *in situ* hydrogel systems are subjected to the trigger approaches in the process of converting the precursor into a solid gel structure. To date, the trigger approaches of smart responsive *in situ* hydrogel systems are achieved by exogenous or endogenous stimuli. The exogenous stimuli strategies require an extra-activation instrument in the process of forming a solid 3D network hydrogel, such as ultraviolet (UV), near-infrared (NIR), or ultrasound (US) (Li et al., 2017). The endogenous stimuli strategies mainly depend on the interaction of both physical microenvironments and precursors (e.g., temperature, pH, enzyme, etc.) (Kumar and Mohammad, 2011; Cheng et al., 2016; Gong et al., 2017). To achieve the various smart responses, the various natural or synthetic hydrogels are usually modified by functional groups (e.g., unsaturated bond) or compounded with functional nanoparticles (e.g., MoS_2_, Cu_2_O, F_3_O_4_, etc.) (Faustino et al., 2023; Wihadmadyatami et al., 2023; Hong et al., 2024).

**FIGURE 1:**
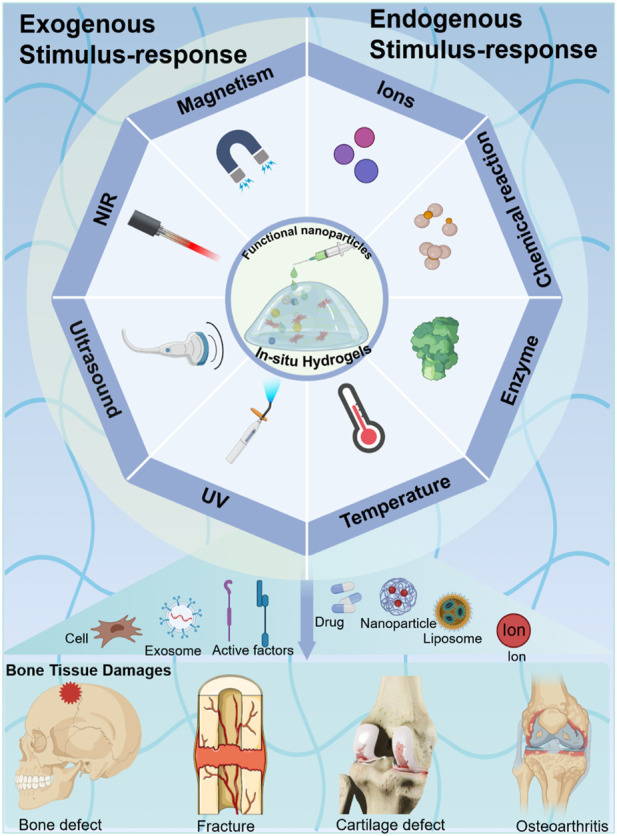
Different stimulus-responsive *in situ* hydrogel systems based on biomaterials were explored to obtain excellent treatment in bone tissue engineering. Exogenous stimulus-responsive systems form *in situ* hydrogels in response to triggers such as UV, NIR, US, etc. Endogenous stimulus-responsive systems form *in situ* hydrogels in response to internal triggers such as temperature, enzyme, etc.

This review provides a brief overview of the various triggers of *in situ* hydrogels in smart crosslinking hydrogel administration and bone tissue regeneration (Figure 1). The various conceptions of trigger approaches for *in situ* hydrogels in bone tissue engineering are highlighted and commented on. In this work, we first introduce the bone tissue structure and microenvironment. Then, we summarize specific and distinct responses to various external stimuli (e.g., UV, NIR, US, etc.) by *in situ* formed hydrogels, which modified biocompatible materials by various functional groups or hybrid functional nanoparticles. Furthermore, we summarize smart hydrogels that respond to physiological environmental stimuli (e.g., temperature, enzyme, etc.) to form an *in situ* delivery system in bone tissue. Lastly, we devote this review to describing the various injectable *in situ* hydrogel systems and their applications in bone tissue. We hope that this review can provide a basic guide for further development of smart responsive *in situ* hydrogels in bone tissue engineering and bone organoid culturing.

## 2 Bone tissue structure and bone tissue microenvironment

Bone is a hierarchical hard tissue that is mineralized and neurovascularized, supports the skeleton, and has many functions, including immunoregulation, growth, movement, hematopoiesis, and organ protection (Sun W. et al., 2023; Wang P. et al., 2024). The homeostasis regulation of bone tissue is primarily relayed in the microenvironment of the ECM. The bone microenvironment is a complex dynastic system composed of a specific ECM, integrins, growth factors, and functional cells (e.g., hematopoietic lineage cells, mesenchymal lineage cells, etc.) (Li Y. Y. et al., 2023; Shi et al., 2023). By gaining a deep understanding of this special biological and physical environment of bone tissue, we can draw inspiration to design more appropriate bone tissue regeneration approaches and promote the bone-healing process.

### 2.1 Bone tissue structure

**FIGURE 2:**
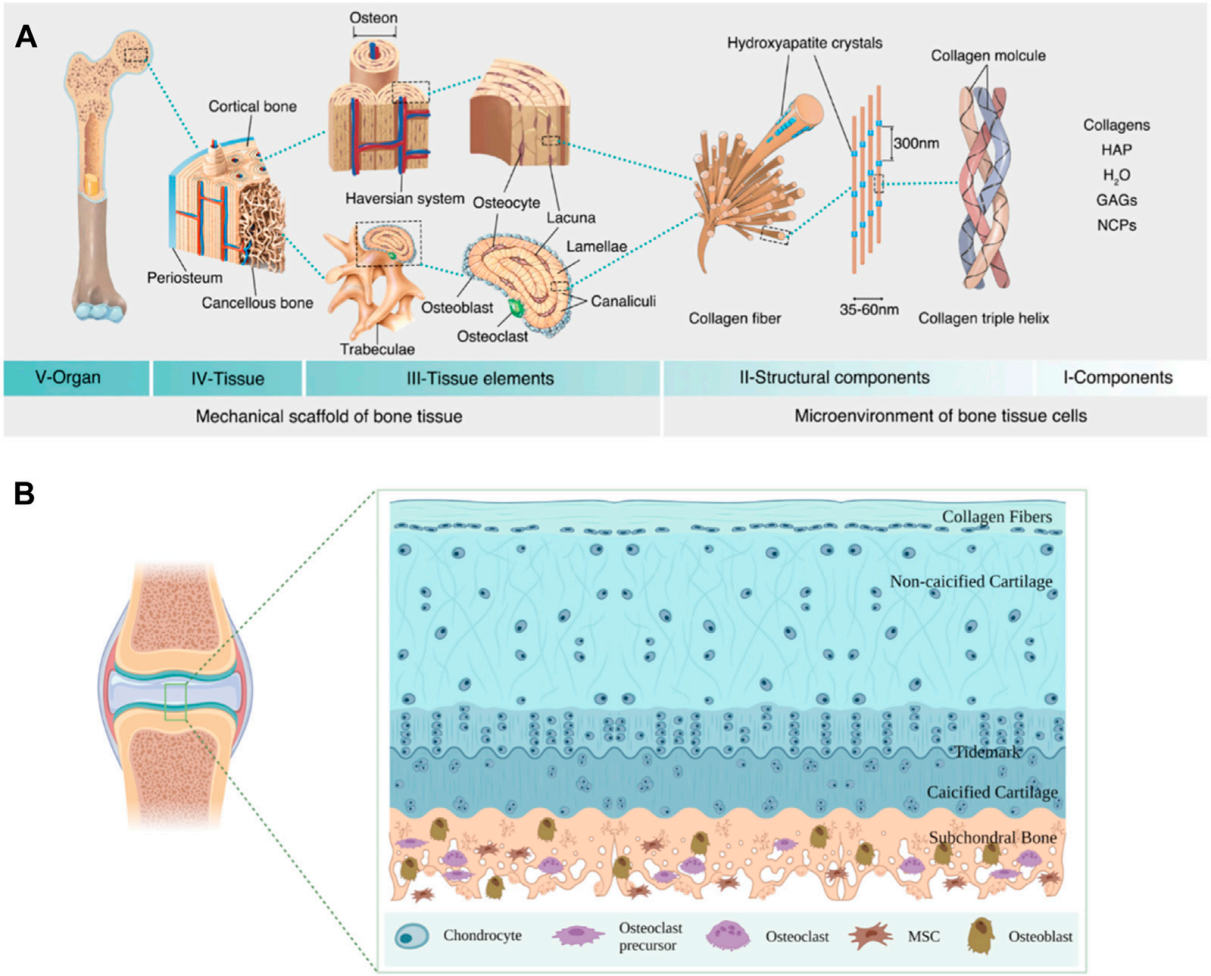
Schematic diagram of bone tissue structure. **(A)** Bone hierarchical structure (Zhu et al., 2021). HAP, hydroxyapatite; GAGs, glycosaminoglycans; NCPs, non-collagenous proteins. Copyright 2021, Elsevier. **(B)** Schematic diagram of cartilage and subchondral bone.

Bone tissue is a highly dynamic and complex structure, which is constantly updated and iterated via the balance of the osteoblasts and osteoclasts (Rodolfi et al., 2023). The structure of bone primarily includes the periosteum, sclerotin, and bone marrow (Abdalla and Pendegrass, 2023). The periosteum is made up of fibrous connective tissue, which is rich in nerves and blood vessels and plays a vital role in bone nutrition, regeneration, and sensation (Caffarelli et al., 2023). Sclerotin is a hierarchical and anisotropic structure, which is mainly composed of dense cortical bone and spongy cancellous bone (Delawan et al., 2023; Tang et al., 2023). The dense cortical bone possesses high mechanical strength and encompasses the cancellous bone on the periphery, which possesses an excellent stabilizing and supporting function. The sponge-like cancellous bone consists of countless flaky trabeculated bones, which are laid out inside the bone. Bone marrow is present in the spongy cancellous bone space and the bone marrow cavity of long bones and is composed of various types of cells and reticulated connective tissue (Caffarelli et al., 2023; Cao et al., 2024). Thus, the bone tissue can be precisely divided into multiple levels for investigation. For example, at the micron level, the cortical bone is arranged along the long axis of the bone. An osteon is the functional basic unit of the long bone, consisting of lamellar bones arranged in concentric circles. Nerve fibers and blood vessels pass through the bones to form the Havers system. The cancellous bone, an anisotropic arrangement of rod-like trabecular bones, forms a honeycomb-like network. Observing the microscopic structure, the main component of ECM is collagen fibers, which have a diameter of approximately 35–60 nm. The collagen fibers are formed by the self-assembly of unique triple-helix biomolecules (Yin and Li, 2006; Wegst et al., 2015; Zhu et al., 2021). Hydroxyapatite crystals with superior anisotropic mechanical properties play a vital role during controlled bio-mineralization (Figure 2A).

### 2.2 Cartilage and subchondral bone

Cartilage is a powerful gliding tissue that covers the moving extremities of the bone skeleton. However, cartilage regeneration is challenging due to its limited ability to self-heal, owing to complex structures and components and a lack of blood vessels (Zhang et al., 2023). Cartilage primarily consists of chondrocytes and ECM (mainly composed of water and type II collagen fibers) (Sun L. et al., 2023). Furthermore, the cartilage defects may initiate the development of osteoarthritis (OA). One work demonstrated bottom-up primary degeneration from subchondral bone during cartilage erosion in OA with aging (Wang H. et al., 2024). The special relationship between subchondral bone and cartilage via various molecular signaling has been widely reported (Wu Z. et al., 2023; Ouyang et al., 2023). For example, the subchondral bone builds a bridge between cartilage and bone to provide both mechanical and nutritional support for cartilage and maintain the stability of the cartilage microenvironment, which is the bony layer below the hyaline cartilage and cement line (Luo et al., 2024). Anatomically, the subchondral bone consists of the subchondral plate and the subchondral bone trabeculae. The subchondral plate is a dense and crisscrossed poly-porous calcified plate. The subchondral bone trabeculae possess a spongy-like cancellous structure behind the subchondral plate, which contributes to continuous bone regeneration and remodeling and maintaining the physiological homeostasis microenvironment of the cartilage (Su et al., 2023; Yu et al., 2023; Zhao et al., 2024) (Figure 2B).

## 3 Application of exogenous stimulus-responsive *in situ* gelling in bone tissue engineering

**FIGURE 3:**
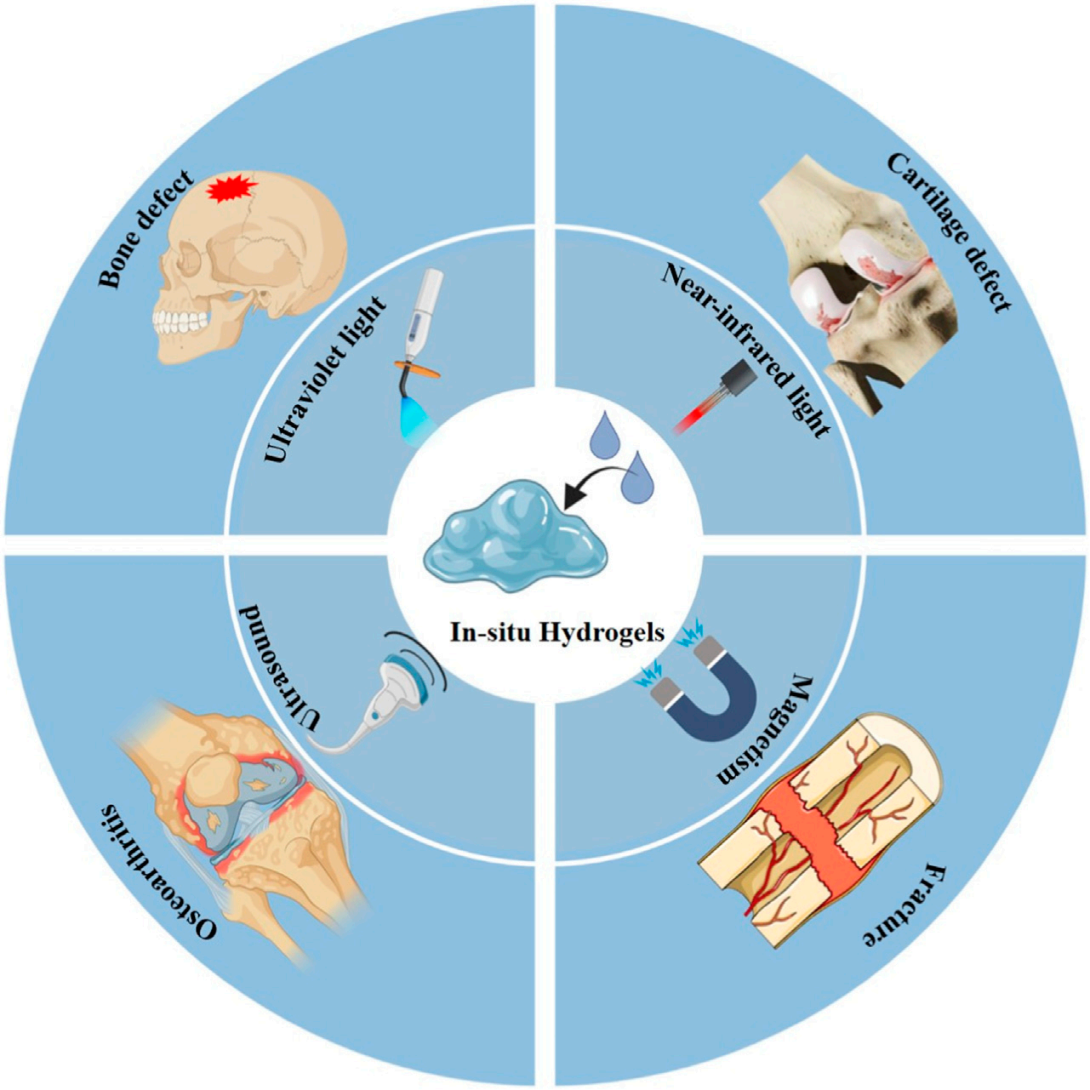
Scheme of various exogenous stimulus strategies for *in situ* hydrogel triggering in bone tissue engineering.

The ideal candidate biomaterials can bridge tissue damage and improve regeneration by enhancing new natural tissue regeneration (Xue et al., 2021). Simultaneously, they are also required to demonstrate excellent biocompatible, tunable mechanical properties and biodegradation as they degrade gradually during tissue repair (Wu D. et al., 2024; Zhou H. et al., 2024; Vaidya et al., 2024). To date, a variety of natural and synthetic biomaterials have been applied in an attempt to simulate ECM of the natural bone tissue (Xu et al., 2023; Sung et al., 2024; Yoon et al., 2024). Hydrogels are regarded as the leader in biomedical and tissue regeneration engineering applications, owing to their 3D structure and characters (Xue et al., 2022b). However, the extensive clinical application of hydrogels faces significant challenges in matching the complex functional and pathological alterations of the human body. Therefore, novel hydrogel biomaterials with smart properties to promote clinical practices in biomedicine and tissue regeneration engineering are needed. Smart responsive *in situ* hydrogels are extensively applied in bone tissue engineering (Ali et al., 2024; Asadi et al., 2024; Zhou H. et al., 2024; Garg et al., 2024). This strategy can guide hydrogels to adapt to diverse shapes and depths of bone defects, which will promote integration with bone tissue. Among the various fabrication strategies, an exogenous stimulus is usually selected to fabricate *in situ* hydrogels for bone repair because of easy control and manipulation via adjusting the input energy flow density. This section mainly reviews the various exogenous stimulus phase transition strategies used for *in situ* hydrogel applications in bone tissue engineering (e.g., UV, NIR, etc.), as shown in Figure 3.

### 3.1 UV light crosslinking to *in situ* hydrogel

Photo-sensitive hydrogels have the ability to achieve a liquid-gel transition when exposed to special wavelengths of light stimuli (Patel et al., 2022). They usually include polymeric systems and functional photoreceptive parts, in which the physicochemical properties are altered (liquid-gel) after light exposure. They have multiple biomedical applications and mainly utilize visible light. Photo-responsiveness is an attractive stimulus method because of its advantages (e.g., light is non-invasive and allows remote control of materials without byproducts). In addition, the light could be precisely modulated by irradiation parameters (e.g., light intensity, irradiation time, input power) to regulate the physical properties of the gels.

**FIGURE 4:**
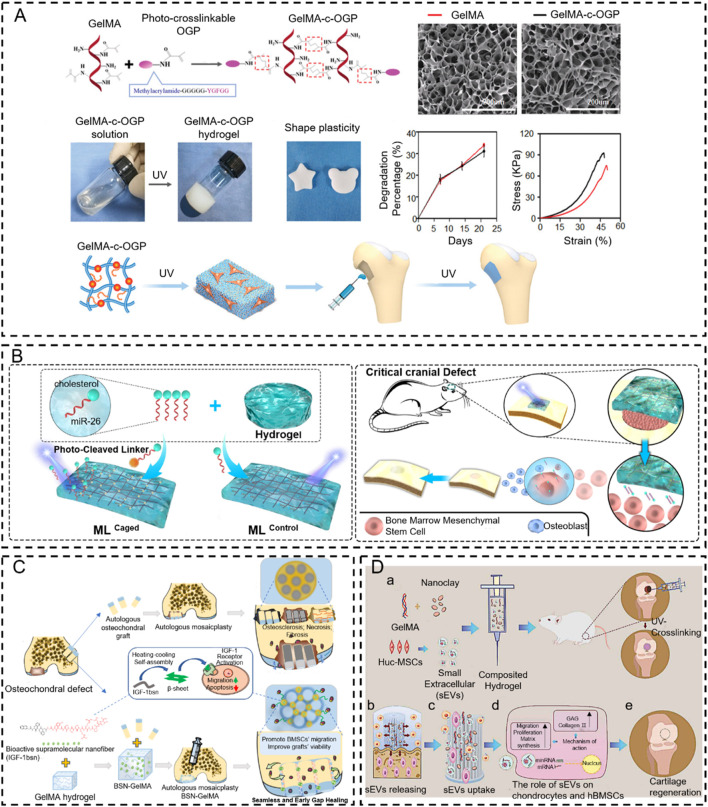
Photo-crosslinking *in situ* hydrogel applied to bone tissue engineering. **(A)** Schematic representation of GelMA-c-OGP hydrogel construction and its mechanical properties (Qiao et al., 2020). Copyright 2020, Wiley-VCH. **(B)** Schematic illustration of bone regeneration *in vivo* at the time of injection of this hydrogel modeled with cholesterol-modified miR-26a at the site of bone malformation (Gan et al., 2021). Copyright 2021, Elsevier. **(C)** Schematic diagram of the IGF-1bsn functioning as activators for promoting cell proliferation and inhibiting apoptosis (Wu H. et al., 2023). Copyright 2023, Elsevier. **(D)** Schematic illustration of therapeutic sEVs released from GelMA/nanoclay hydrogel for cartilage regeneration (Hu et al., 2020). Copyright 2020, Taylor & Francis Group.

However, most natural biomaterials fail to respond to the trigger condition due to the absence of photo-responsive functional groups (Ding et al., 2015). Fortunately, these *in situ* response systems are achieved by introducing functional groups through photochemical addition reactions or activation of functional groups via the release of photo-caging groups and photoisomerization processes. Some bioactive materials are modified via methacryloyl (MA) or unsaturated bond groups (C=C or clickable C≡C) to respond to light triggers (e.g., gelatin methacryloyl (GelMA) (Zhang et al., 2016; Byambaa et al., 2017; Sun X. M. et al., 2018), hyaluronic acid- butyramide (HA-NB) (Zhang et al., 2016), polyethylene glycol (PEG) (Burke et al., 2019), etc.). For example, Qiao et al. (2020) designed a novel injectable *in situ* osteogenic polypeptide hydrogel system (GelMA-c-OGP) via ultraviolet radiation co-crosslinking with photo-cross-linkable osteogenic growth peptides (OGP). The *in situ* hydrogel system accelerates the bone regeneration process of osteogenic precursor cells by reinforcing the expression of osteogenic-related genes and increasing the precipitation of calcium in osteoblasts (Qiao et al., 2020) (Figure 4A). The injectable hydrogel precursor converts to solid hydrogel with UV *in situ* irradiation, which also can reinforce the mechanical properties (e.g., stress-strain, shear force.) of the defective bone, avoid burst release osteogenic drugs, and achieve timed release throughout the bone-healing term. Similar work was reported by Montazerian, H. to enhance the toughness, stretchability, and adhesive properties of GelMA hydrogel. They conjugated the polydopamine to the GelMA to improve the cell migration capacity and enhance bone regeneration (Montazerian et al., 2021). Furthermore, researchers have recently focused on RNA treatment due to its high effectiveness. For example, Gan et al. (2021) utilized the cholesterol-modified non-coding microRNA chol-miR-26a to promote the osteogenic differentiation of human mesenchymal stem cells (hMSCs). Chol-miR-26a was conjugated to an injectable poly(ethylene glycol) (PEG) hydrogel by an ultraviolet (UV)-cleavable ester bond. The injectable PEG hydrogel was formed by a copper-free click reaction between the terminal azide groups of 8-armed PEG and dibenzocyclooctyne-biofunctionalized PEG, while UV-cleavable chol-miR-26a was simultaneously conjugated via a Michael addition reaction. Upon UV irradiation, Gel-c-miR-26a (ML^Caged^) not only released chol-c-miR-26a selectively but also exhibited a significantly improved efficacy in bone regeneration compared to the hydrogel without UV irradiation and UV-uncleavable ML^Control^ (Gan et al., 2021) (Figure 4B).

In addition to traditional bone regeneration, the UV-mediated *in situ* hydrogel is combined with an autologous bone grafting technique to improve osseointegration and bone fusion. For example, Wu et al. (Wu H. et al., 2023) initiated a new method to mend the gaps between osteochondral plugs after mosaicplasty. This approach employs an injectable photo-sensitive hydrogel (BSN-GelMA), which is fabricated by GelMA loaded with bioactive supramolecular nanofibers to mimic IGF-1(IGF-1bsn) that can bind to IGF-1 receptors and improve the bioavailability, due to their high stability and tissue retention. The result demonstrated that the BSN-GelMA could realize seamless osteochondral integration in the gap region between plugs of osteochondral defects after mosaicplasty because they can prevent cartilage degeneration and promote graft survival. These bio-interactive materials offer a way to bridge the gap and enhance osteochondral integration after mosaicplasty (Figure 4C). Recently, UV-mediated *in situ* hydrogel became an ideal strategy for improving cartilage regeneration (Huang et al., 2018; Xue et al., 2022a). Articular cartilage has limited self-regenerative capacity, and the therapeutic methods for cartilage defects are still dissatisfactory. To overcome this problem, Hu et al. (2020) fabricated a GelMA/nanoclay hydrogel medium for sustaining the release of extracellular vesicles that come from human umbilical cord mesenchymal stem cell-derived small extracellular vesicles (hUC-MSCs-sEVs) to improve the cartilage regeneration, which exhibited outstanding mechanical property. The results revealed that the GelMA/nanoclay hydrogel containing hUC-MSCs-sEVs stimulated chondrogenesis and healed cartilage damage by improving the expression of collagen II and glycosaminoglycan (GAG) (Figure 4D).

Although photo-sensitive hydrogels possess high sensitivity and are widely applied in research, there are several shortcomings that need to be considered in clinical application. Due to the limited ability of ultraviolet light to penetrate tissue, photoactivation must be directed *in situ* after gel injection, which is difficult in clinical practice. Moreover, there is a need for additional chemical reactions to graft special functional groups due to the most natural biomaterials do not have triggers for photoactivation. This complicates the preparation of photo-sensitive hydrogels and makes industrial production more difficult. Therefore, the production and preparation of photo-responsive gel precursors with high safety can improve the clinical practice of UV-stimulated *in situ* hydrogels.

### 3.2 NIR crosslinking to *in situ* hydrogels

**FIGURE 5:**
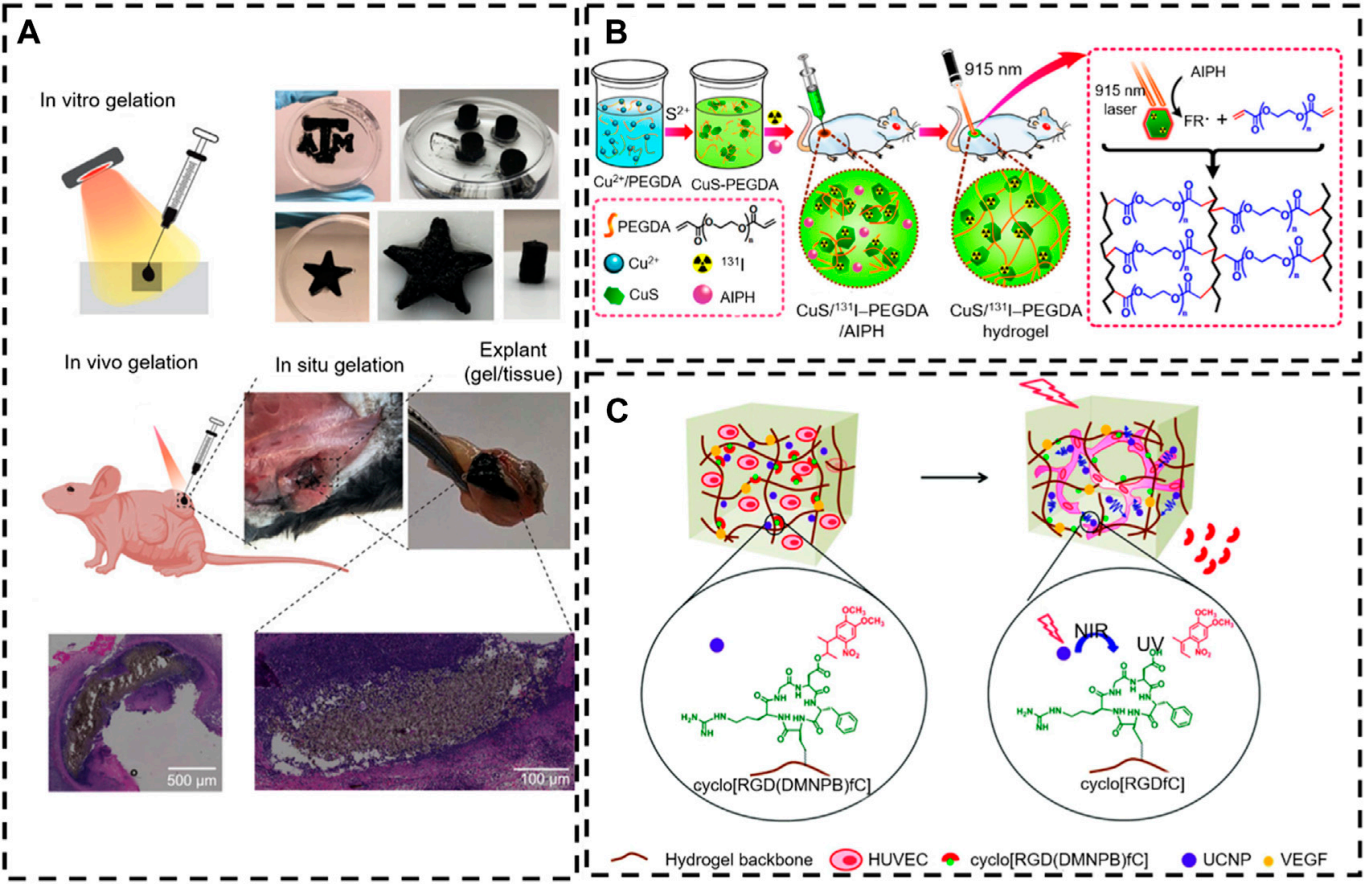
NIR-responsive *in situ* hydrogel in tissue engineering. **(A)** *In situ* gelation *in vitro* and *in vivo* of PNAM–MoS_2_ (Lee et al., 2021). Copyright 2021, Wiley-VCH. **(B)** The controllable *in situ* synthesis of a CuS/^131^I -PEGDA hydrogel (Meng et al., 2018). Copyright 2018, American Chemical Society. **(C)** Schematic illustration of NIR light-induced angiogenesis in a photoactivatable hydrogel with embedded UCNP-PMAOs (Zheng et al., 2020). Copyright 2021, RSC.

Near-infrared (NIR) is an electromagnetic wave between visible light (VI.S) and mid-infrared light (MIR) and has a wavelength in the range of 780–2,526 nm as defined by the American Association for Testing and Materials Testing (ASTM) (Zhang et al., 2024c). The NIR spectrum belongs to the frequency multiplier and main frequency absorption spectrum of the molecular vibration spectrum due to the non-resonance of the molecular vibration (the molecular vibration is generated when the molecular vibration transitions from the ground state to the high energy level). The NIR possesses a strong penetration ability because of its special physical properties (Wang K. et al., 2024). In addition, NIR, without being strongly absorbed by body fluid, presents better tissue transparency than other wavelengths and is an ideal candidate for designing light-responsive *in situ* hydrogels (Raza et al., 2019). Thus, many injectable hydrogel systems have been explored to construct an *in situ* hydrogel in niduses via an NIR light trigger due to its noninvasive nature, high spatial resolution, temporal control, and convenience (Abueva et al., 2020) (Figure 5).

The photothermal effect is the primary mechanism of NIR-responsive *in situ* hydrogels. Precisely, it refers to the release of vibrational energy (heat) while the nanoparticle (NP) photosensitizer is excited by a specific band of light in the precursor (the conversion of light to heat) and then forms a hydrogel via the tune temperature (Kim and Lee, 2018). The NIR light was applied as an “on/off” trigger to remotely heat and activate thermosensitive *in situ* hydrogels. Similar systems are used for on-demand drug delivery and synergistic photothermal chemotherapy. For example, Liu et al. (2019) designed an injectable, thermosensitive photothermal-network hydrogel (PNT-gel) through host-guest self-assembly of photothermal conjugated polymers and ɑ-cyclodextrin. The conjugated polymer backbones can directly convert incident light into heat, endowing the PNT-gel with high photothermal conversion efficiency (g = 52.6%) and enhancing photothermal stability. Meanwhile, the mild host-guest assembly enables shear-thinning injectability and the photothermally driven and reversible gel-sol conversion of the hydrogel.

Many nanoparticles with photothermal conversion properties (including nanostructures of Au, Ag, Pt, Pd, graphene oxide, carbon nanotubes, CuS, MoS_2_, and PDA) have been developed to work as multifunctional nanoplatforms to realize photothermal therapy in tissue engineering (Jiang et al., 2019). Lee et al. (2021) designed a novel type of NIR-triggered *in situ* gelation system based on the defect-rich 2D MoS_2_ nano-assemblies and thiol-functionalized thermos-responsive poly (N-isopropyl acrylamide-co-acrylamide-co-2-mercaptoethylacrylamide) (PNAM). In this process, the dynamic polymer–nanomaterial interactions activate under the NIR radiation without a photoinitiator due to the photothermal characteristics of MoS_2_ and the intrinsic phase transition ability of the PNAM thermos-responsive polymer. Specifically, this phase transition behavior of PNAM stems from dynamic changes in the hydrophilic properties of the lateral radicals. Thus, as the temperature increases, the hydrophobic interaction of MoS_2_ nanoparticles increases with the PNAM chain. In addition, the MoS_2_ nanoparticles generate heat under near-infrared irradiation to attract vulcanized PNAM chains to wrap themselves, while the defects of MoS_2_ nanoparticles react rapidly with the thiol side groups to form covalent bonds (Figure 5A). Consequently, the remotely controlled on-demand release of drugs is achieved via a photothermal-induced gel-sol transition (Wu et al., 2017; Meng et al., 2018; Wang et al., 2020) (Figure 5B).

**FIGURE 6:**
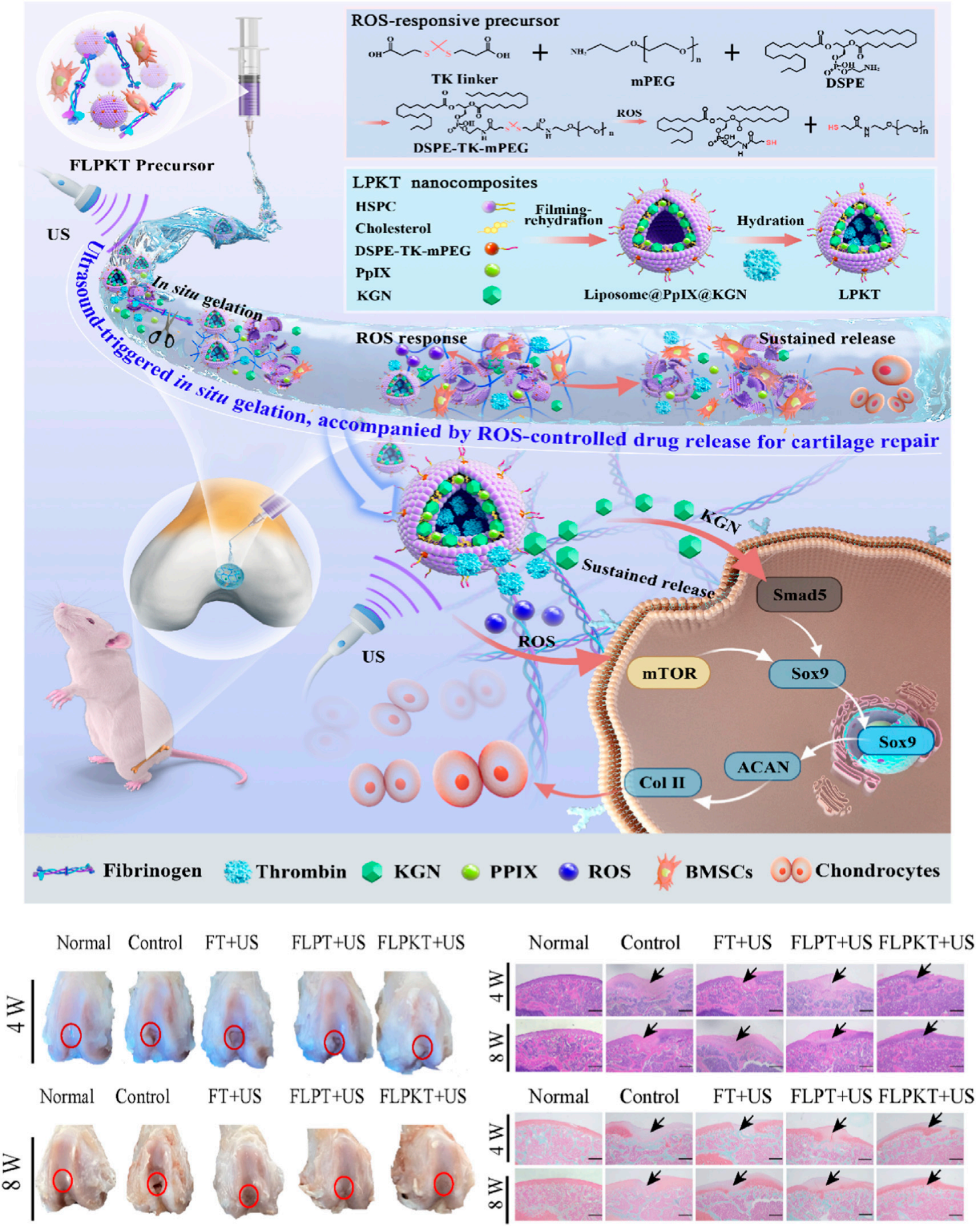
Schematic illustration of the design of *in situ* nanocomposite hydrogels with a controlled release of KGN and ultrasonic stimuli ROS production for irregular cartilage repair (Wu S. et al., 2023). Copyright 2023, RSC.

The photothermal effect of the incorporated photothermal agents not only induced the on-demand drug release through the gel-sol phase transition of thermosensitive hydrogel but also induced proliferative differentiation of cells via hyperthermia (Shen et al., 2021). Here, Zheng et al. (2020) described an NIR strategy for controlling activated cell processes (3D cell diffusion and angiogenesis) by embedding up-conversion nanoparticles (UCNPs) into the hydrogel, which is modified with a photo-activated cell adhesion motif. The UCNPs can convert NIR light (974 nm) into localized UV emission and activate photochemical reactions on demand. This light regulation is spatially controllable and dose-dependent and can be performed at different time points in cell culture without causing significant photodamage to the cells. Human umbilical vein endothelial cells (HUVEC) cells embedded in this hydrogel can form a network of blood vessels at predefined geometries determined by the irradiation pattern (Figure 6C). There are two main mechanisms of *in situ* hydrogel gel formation induced by NIR. The first is to realize photothermal conversion through a photothermal factor, and the other is to convert near-infrared light into ultraviolet light by UCNPs and then induce *in situ* gelling. However, the NIR utilization rate of both types of gelatinization is very low.

### 3.3 US-responsive *in situ* hydrogel

Ultrasound is a potentially valuable trigger for hydrogels because of the mechanical pressure waves that oscillate at high frequencies (≥20 kHz) and produce a range of thermal and nonthermal effects (Wiklund et al., 2012). The thermal effects refer to the transition from acoustic energy to thermal energy, inducing a rise in temperature in tissues (Gao et al., 2005). The nonthermal effects, also named cavitation effects, refer to the tiny gas bubbles generated by ultrasound vibration (Manouras and Vamvakaki, 2017).

Ultrasound can trigger reactions in polymers, which has been reported since the 1930s (Price, 2003). This system enables noninvasive, spatiotemporally controlled modulation and morphological properties using focused ultrasound in the body. For example, Wu S. et al. (2023) designed and prepared a novel ROS-responsive controlled-release *in situ* drug nanohydrogel for articular cartilage repair (Figure 6). Ultrasonic-cleavable ketothiol (TK)-based nanoliposomes are loaded into cartilage drugs (KGNs), thrombin, and sonics (PpIX). Partial rupture of liposomes under ultrasound stimulation is accompanied by enzymatic reactions of fibrinogen and thrombin to achieve *in situ* gelation for filling tissue defects. In addition, the controllable release of KGN drugs and the appropriate amount of ROS production were achieved by regulating the ultrasound conditions. More importantly, the appropriate amount of ROS and the continuous release of KGN in the *in situ* nanocomposite hydrogel microenvironment effectively promoted the differentiation of BMSCs in the direction of cartilage formation by stimulating the Smad5/mTOR signaling pathway, thereby accelerating the repair of articular cartilage defects. Current research also focuses on inducing *in situ* gelatinization through cascade reactions. The development of ultrasound *in situ* hydrogels is slow due to the lack of ultrasound responsiveness of natural gel materials. There is still a long distance to clinical application.

### 3.4 Magnetic responsive *in situ* hydrogel

Magnetic hydrogels (MHGs) have extensive tissue engineering and biomedical applications and possess the advantages of quick response, biocompatibility, tunable mechanical properties, porosity, and internal morphology (Asadi et al., 2024; Liu et al., 2024). MHGs can be fabricated by blending exogenous additives (e.g., paramagnetic, ferromagnetic) in the polymeric matrix and placing the matrix in a magnetic field (static or oscillating) (Zhou K. et al., 2024; Zhang K. et al., 2024).

The most common and straightforward method for making magnetic composite hydrogels is blending with magnetically responsive nanoparticles. For example, Fe_3_O_4_ is one of the most common magnetic nanoparticles (MNPs) mixed with a polymer for the preparation of magnetic hydrogels. The Fe_3_O_4_ magnetic nanocomposite gel can be adjusted by applying a magnetic field (Yan et al., 2022). In addition, the type of hydrogel networks and MNPs (the content, size, distribution), as well as the interactions between MNPs and polymer networks, can make a significant impact on the physicochemical properties and magnetic responsiveness of magnetic hydrogels (Thoniyot et al., 2015). Magnetite and maghemite have been prepared and loaded into dextran hydrogel to produce a magneto-sensitive composite hydrogel system by photopolymerization (Brunsen et al., 2012). The *in situ* synthesis of MNP-based composite hydrogels is more accurate in terms of particle size and hydrogel architecture (Farzaneh et al., 2021). Gao et al. (2014) developed a magnetic hydrogel composed of anion networks of poly (2-acrylamide-2-methylpropane sulfonic acid) (PAMPS) and anisotropic nanocrystals of Fe_3_O_4_. The Fe_3_O_4_ nanocrystals were prepared *in situ* in the PAMPS networks, where negatively loaded SO_3_ facilitated the adsorption and uniform distribution of iron ions in the hydrogel network. They found that the size and shape of Fe_3_O_4_ nanocrystals can be adjusted by modifying the crosslinking density of hydrogel without applying stringent experimental conditions (Gao et al., 2014). Although the magnetic response *in situ* hydrogel has achieved excellent results, it still faces great challenges in the clinical transformation stage due to the complex composition and the need to introduce magnetic response factors. Therefore, simplifying the composition of magnetic response *in situ* hydrogels and increasing their usability will be conducive to their use in tissue engineering.

## 4 Application of endogenous stimulus-responsive *in situ* hydrogels in bone tissue engineering

Endogenous stimulus-responsive *in situ* hydrogels with no additional stimulus approaches and easy control have been extensively applied in tissue engineering (e.g., temperature, enzyme, etc.) under physiological and pathologic environment conditions (Wu P. et al., 2024; Gang et al., 2024). In particular, these stimuli-sensitive hydrogels can exhibit significant sol-to-gel transitions after certain stimuli. Hydrogels with appropriate reactivity are synthesized for different practical applications, such as modulation of cargo release and/or replication of dynamic interactions/evolutions in the *in situ* microenvironment (Yoon et al., 2012).

### 4.1 Temperature stimulus-responsive *in situ* hydrogel

Amongst several stimuli-responsive *in situ* hydrogel systems, the thermal stimulation *in situ* hydrogel is one of the most widely investigated classes because they have a lower critical solution temperature (LCST) and can flexibly modify their physical characteristic (e.g., changes in volume) in response to alters in various temperatures (Ma et al., 2019; Li X. et al., 2021; Wang J. et al., 2021; Nguyen et al., 2021). Thermo-gel behavior combines different thermosensitive mechanisms, such as the precipitation of water-insoluble polymers above LCST and then the aggregation of thermo-gelling (Zhang K. et al., 2021). Precisely, the hydrogel precursor is liquid when kept at temperatures lower than the critical solution temperature and transforms into solid-phase gel at body physiological temperature (Zhang et al., 2019). This smart strategy can form an *in situ* hydrogel and offer the possibility of injecting those systems and conforming to any irregular defects (El-Husseiny et al., 2022).

Many kinds of thermo-responsive hydrogels have been reported to improve the regeneration of bone tissue. Temperature-responsive hydrogels usually possess distinctive thermoplastic characteristics owing to hydrophobic moieties (e.g., propyl, ethyl, methyl group, etc.) (Sood et al., 2016). For example, poly (N-isopropyl acrylamide) (PNIPAm) gel has an obvious coil–globule transition at 32 °C (LCST); the compound is hydrophilic below this temperature and hydrophobic above it. Thus, these injectable precursors show a solution state at ambient temperature; the gel form is produced *in situ* at physiologic temperature (Karimi et al., 2016). Many thermo-responsive hydrogels based on PNIPAms have been designed and applied in tissue engineering, including N-isopropyl acrylamide hydrogel crosslinked with di(ethylene glycol) divinyl ether poly (NIPAAm-co-DEGDVE) (Werzer et al., 2019) and polymer poly [(propylene sulfide)-block-(N,N-dimethyl acrylamide)-block-(N-isopropyl acrylamide)] (PPS-b-PDMA-b-PNIPAAM) (Gupta et al., 2014).

**FIGURE 7:**
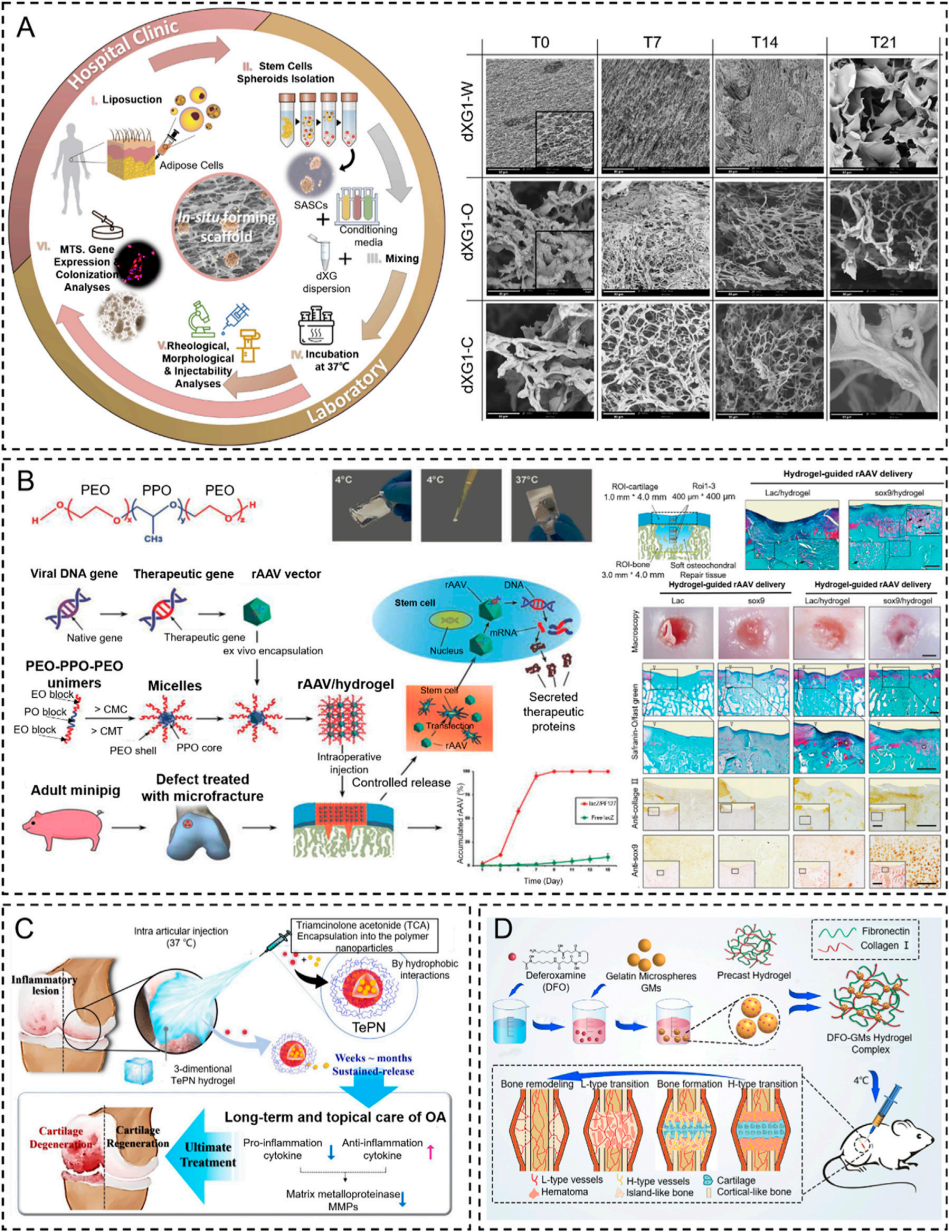
Temperature stimulus-responsive *in situ* hydrogel applied in bone tissue engineering. **(A)** Graphical representation of the injectable *in situ* hydrogel experimental strategy *in vivo* or *in vitro* (Muscolino et al., 2021). Copyright 2021, Elsevier. **(B)** Schematic representation of PF127-based copolymer temperature stimulus-responsive *in situ* hydrogel in chondral regeneration (Madry et al., 2020). Copyright 2020, Wiley-VCH. **(C)** Schematic diagram of one-time injection of the TePN hydrogel system for long-term osteoarthritis treatment (Seo et al., 2022). Copyright 2022, Elsevier. **(D)** Preparation of DFO-GMs hydrogel complex for repairing a critical-sized femoral defect (Zeng et al., 2022). Copyright 2022, Elsevier.

Poloxamers are another biodegradable biomaterial that has been widely applied to thermo-responsive *in situ* hydrogel systems in tissue engineering due to the suitable sol-gel transition temperature (Liu et al., 2020; Zhang T. et al., 2021). For example, Madry et al. (2020) designed an injectable and thermosensitive hydrogel based on poly(ethylene oxide) (PEO)–poly(propylene oxide)(PPO)–PEO poloxamers, capable of controlling the release of a therapeutic recombinant adeno-associated virus (rAAV) vector overexpressing the chondrogenic Sox9 transcription factor in full-thickness chondral defects. This work indicated that the rAAV-FLAG-hsox9/PEO–PPO–PEO hydrogel significantly improves cartilage repair with a collagen fiber orientation similar to the normal cartilage and protects the subchondral bone plate from early bone loss in a minipig model (Madry et al., 2020) (Figure 7A).

Several natural biomaterials (e.g., cellulose and gelatin) and artificial synthetic polymers (e.g., poly (N-isopropyl acrylamide) (PNIPAAm), poloxamer, and polyfluorene 127, etc.) are exploited to fabricate thermosensitive hydrogels which suggest promising applications for bone tissue engineering (Chatterjee et al., 2018). For example, Ren et al. (2015) developed a thermosensitive hydrogel by gelatin grafted with PNIPAAm (Gel–PNIPAAm). These injectable hydrogel precursors exhibit an excellent biocompatible, sol-to-gel transformation property at physiological temperature and an excellent fill-ability for complex and irregular bone defects. The result demonstrated that this gel–PNIPAAm thermosensitive *in situ* hydrogel can induce bone differentiation and improve bone regeneration compared to the control in a cranial damage model.

As recently reported, several natural polysaccharides (e.g., agarose, amylose, carrageenan, etc.) possess thermo-gelling properties, but they failed to form gels at body temperature (Zhang B. et al., 2024; Elizalde-Cárdenas et al., 2024; Riviello et al., 2024). Fortunately, when xyloglucan is partially degalactosylated (Deg-XG), it will achieve sol-to-gel at a physiological temperature of concentration over 2 wt% (Chandramouli et al., 2012). For example, E.M and colleagues presented a simple strategy based on the synergic combination of forming *in situ* hydrogels and spheroids of adipose stem cells (SASCs) with great potential for minimally invasive regenerative interventions aimed to treat bone and cartilage defects. They loaded SASCs in aqueous dispersions of partially Deg-XG and either a chondroinductive or an osteoinductive medium to induce bone and cartilage regeneration. The dispersions rapidly set into hydrogels when the temperature was brought to 37°C. The physicochemical and mechanical properties of the hydrogels are controlled by polymer concentration (Muscolino et al., 2021) (Figure 7B). Similar results were reported by Dispenza et al. (2017). They designed the same injectable thermosensitive hydrogel (Deg-XG loading with the growth factor FGF-18) to promote cartilage reconstruction at gelation at 37°C.

Thermosensitive hydrogels were also extensively applied in the effective treatment of osteoarthritis by local injection to replace oral administration. For example, Seo et al. (2022) designed an injectable polymeric nanoparticle hydrogel system with poly(organophosphazenes), which is loaded with triamcinolone acetate (TCA, nonsteroidal anti-inflammatory drugs) for achieving a long-term anti-inflammatory effect and treat osteoarthritis. The TePN precursor turned into a solid hydrogel after intra-articular injection, and the formed 3D TePN hydrogel released TePNs for months in an *in vivo* microenvironment. Long-term release of TCA treats OA by inhibiting matrix proteinase (MMP) expressions in the cartilages via decreased pro-inflammatory and increased anti-inflammatory cytokine expressions (Figure 7C).

Insufficient vascularization is still a great challenge in the regeneration of critical-sized bone tissue defects (Takaoka et al., 2024; Yuan et al., 2024). The formation of the H-type vessel plays a key role in the progress. To address this problem, Zeng et al. (2022) designed an injectable thermosensitive demethoxyamine (DFO) gelatin microsphere (GMs) hydrogel complex to stimulate the production of functional H-type blood vessels. The results showed that the DFO-GMs thermosensitive hydrogel complex could stimulate the production of functional H-type blood vessels and effectively promote the proliferation, formation, and migration of HUVECs *in vitro*. Moreover, this thermosensitive hydrogel can expand the distribution range of H-type blood vessels in the defect area and increase the native bone tissue, which was confirmed via fluorescence immunostaining and radioactivity examination *in vivo* (Figure 7D).

The thermo-responsive hydrogels possess great potential to adjust the threshold response levels and allow on-demand triggers due to excellent biocompatibility, mild trigger conditions, no toxicity, etc. More studies should examine the integration of thermo-responsive hydrogels with multi-gradient temperature triggers. In addition, thermo-responsive hydrogel systems are generally regarded as crucial triggers in designing advanced external stimuli (e.g., NIR, magnetic, US, etc.) for smart, responsive platforms.

### 4.2 Enzyme stimulus-responsive *in situ* hydrogel

**FIGURE 8:**
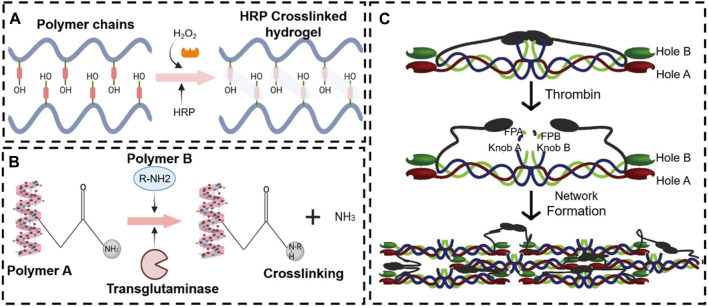
Various enzyme stimulus-responsive *in situ* hydrogel schemes. Enzyme response mechanisms of horseradish peroxidase **(A)**, transglutaminase **(B)**, and thrombin **(C)**.

Biocatalytic reactions of enzyme complexes are especially important in naturally occurring multicellular organisms (Bleier et al., 2015). Furthermore, the enzymes catalyze the recognition of the 3D substrate structures that are adapted to the enzymes for binding. The “substrate specificity” closely regulates enzymatic activity without adverse side effects. In recent years, the enzyme medium stimulus-responsive *in situ* hydrogels have played an increasing role in tissue repair due to the mild reaction mechanism. Most enzymes involved in crosslinking also catalyze natural reactions in our body (Jin et al., 2011) (Figure 8). Furthermore, the enzymatic reactions are catalyzed with a neutral-pH microenvironment in an aqueous medium at moderate temperatures, which means that they can also be utilized to develop *in situ* hydrogels. Smart enzymatic reaction systems can be designed not only to create native extracellular matrices (ECM) but also to construct degradable biomaterials (Hubbell, 2003). These events capture, in substance, one of the most significant biological features of the ECM, which is remodeling. In addition to degradability, it is essential to adapt the gelation rate to applications such as drug administration and tissue regeneration strategies. A controlled gelation rate is essential to prevent diffusion of the precursors, ensure localized drug delivery, obtain a suitable cell distribution, and properly integrate the gel with the surrounding tissues (mainly for irregular-shape filling applications) (Teixeira et al., 2012). The enzyme stimulus-responsive *in situ* hydrogel has raised much attention in bone tissue engineering (e.g., OA, bone defects, fracture, etc.) (Zhou S. et al., 2024; Vidal et al., 2024). In this contribution, the enzymatically crosslinked gels are reviewed in the context of regenerative strategy applications.

#### 4.2.1 Peroxidase

Peroxidases are a broad family of enzymes that usually catalyze the following reaction: ROOR′ + electron donor (2e^−^) + 2H^+^—ROH + R′OH. Horseradish peroxidase (HRP) is the most common peroxide used in the formation of hydrogel (Krainer and Glieder, 2015; Khanmohammadi et al., 2018). HRP is widely distributed in the plant kingdom. It is a colorless enzyme protein comprising brown iron porphyrin combined with glycoprotein. In addition, HRP is combined with hydrogen peroxide (H_2_O_2_) to obtain an [HRP-H_2_O_2_] complex that can oxidize a variety of hydrogel donor substrates (Ryan et al., 2006).

**FIGURE 9:**
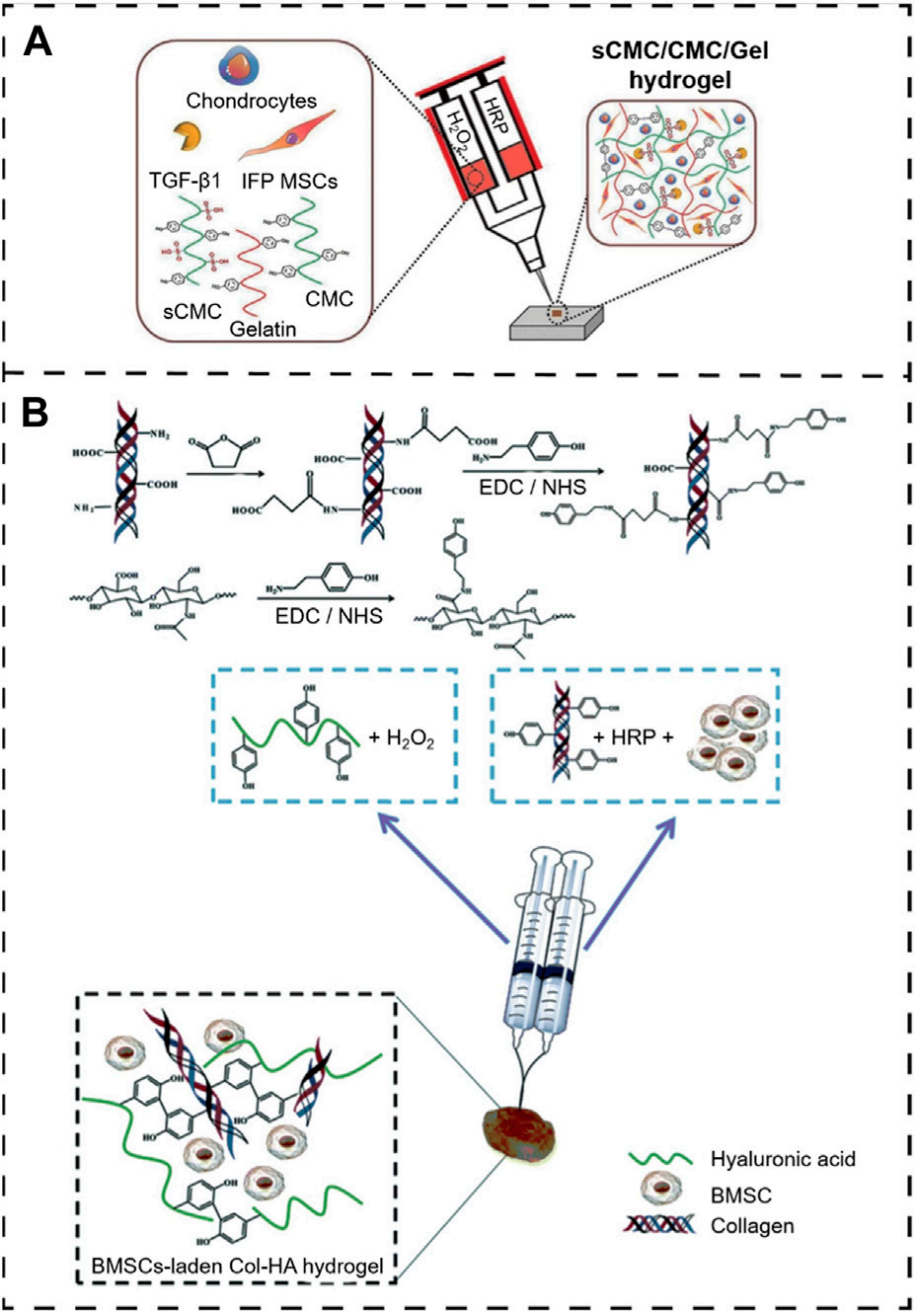
Peroxidase enzymatically crosslinking injectable *in situ* hydrogels. **(A)** Overall scheme of enzymatically cross-linked injectable hydrogels for delivery of cells and TGF-β1 for cartilage tissue engineering (Arora et al., 2017). Copyright 2017, Europe PMC. **(B)** Preparation of the BMSC-laden injectable Col-HA hydrogel for cartilage regeneration (Zhang et al., 2020). Copyright 2020, RSC.

In crosslinking, the basic physical properties (mechanisms) and biologically inducible properties mainly rely on the precursor concentration. Saghati and colleagues (Saghati et al., 2021) detect the effect of several parameters, including H_2_O_2_ and HRP concentrations, on the hydrogel properties of Alg-based hydrogels via an enzymatic cross-linked procedure. They found that the physical properties of 1.2% (v/w) Alg-Ph, 5 U HRP, and 100 mM H_2_O_2_ at the ratio of 1:0.54:0.54 are more appropriate for the cartilage-like structure, which has a great potential to induce cartilage regeneration. Several studies have been reported to describe enzymatic crosslinking *in situ* hydrogels (Arora et al., 2017; Qi et al., 2018; Behrendt et al., 2020; Li Q. et al., 2021). For example, Arora et al. (2017) developed a novel enzymatically cross-linked injectable hydrogel composed of carboxymethyl cellulose (CMC), sulfated carboxymethyl cellulose (sCMC), and gelatin for the delivery of infrapatellar fat pad-derived MSCs and articular chondrocytes to a cartilage defect site while enabling TGF-β1-mediated chondrogenesis (Figure 9A). Similarly, Zhang et al. (2020) fabricated an *in situ* hydrogel consisting of collagen-type I-tyramine (Col-TA) and hyaluronic acid-tyramine (HA-TA) that also loaded the BMSC for cartilage regeneration. Further, the proliferation and differentiation of BMSCs within the Col-HA hydrogel were evaluated, and the ability of *in vivo* cartilage repair was also examined in the presence of the TGF-β1. These results illustrated that this hydrogel could provide an excellent microenvironment for BMSC growth and cartilage differentiation *in vitro* and *in vivo* (Zhang et al., 2020) (Figure 9B).

#### 4.2.2 Per-oxygen transglutaminases

**FIGURE 10:**
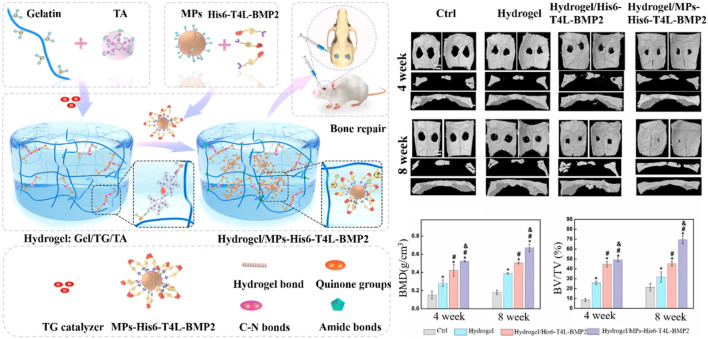
Fabrication of hydrogel Gel/TG/TA-MPs-His6-T4L-BMP2 (Hydrogel/MPs-His6-T4L-BMP2), which was applied to bone defects (Chen X. et al., 2021). Copyright 2021, Elsevier.

Transglutaminase, a type of biological glue, is found in plasma, tissues, keratinocytes, and epidermal cells in the human body. Transglutaminase catalyzes the development of an isopeptide binding of the amide group of a glutamine residue to the primary amine group of a lysine residue (Greenberg et al., 1991). The amide portion of the glutamine residue attacks the enzyme as the donor acrylic substrate (an intermediary thioester form), resulting in an acrylic-enzyme form. In the presence of an acyl-acceptor substrate, such as a lysine residue, the imidazole group in the active site was deprotonated by the amine group, while the acyl-enzyme and lysine residues form a tetrahedral group (Lai et al., 2017; Savoca et al., 2018). Finally, this group decomposes into an isopeptide group to form a reticulated system^78^. In addition, transglutaminase was applied to hydrogel as an enzymatic crosslinking agent to achieve *in situ* gelling, due to the safe and highly effective catalytic effect. For example, Chen X. et al. (2021) manufactured a novel gelatin-based hydrogel that was crosslinked by transglutaminase (TG) and tannic acid (TA). This hydrogel, when incorporated into the MPs-His6-T4L-BMP2, demonstrated excellent *in situ* injectability, heat sensitivity, adhesion, and mechanical properties. The efficient charging mode resulted in a controllable and sustainable release of His6-T4L-BMP2 to improve bone regeneration in a critical bone defect (Figure 10). The injection and tunable hyaluronan-transglutaminase hydrogel were obtained by adding coagulation factor XIII. The specific amino acid residues from substrate FXIIIa contribute to reducing toxicity during crosslinking (Anjum et al., 2016).

#### 4.2.3 Fibrin-thrombin hydrogel

**FIGURE 11:**
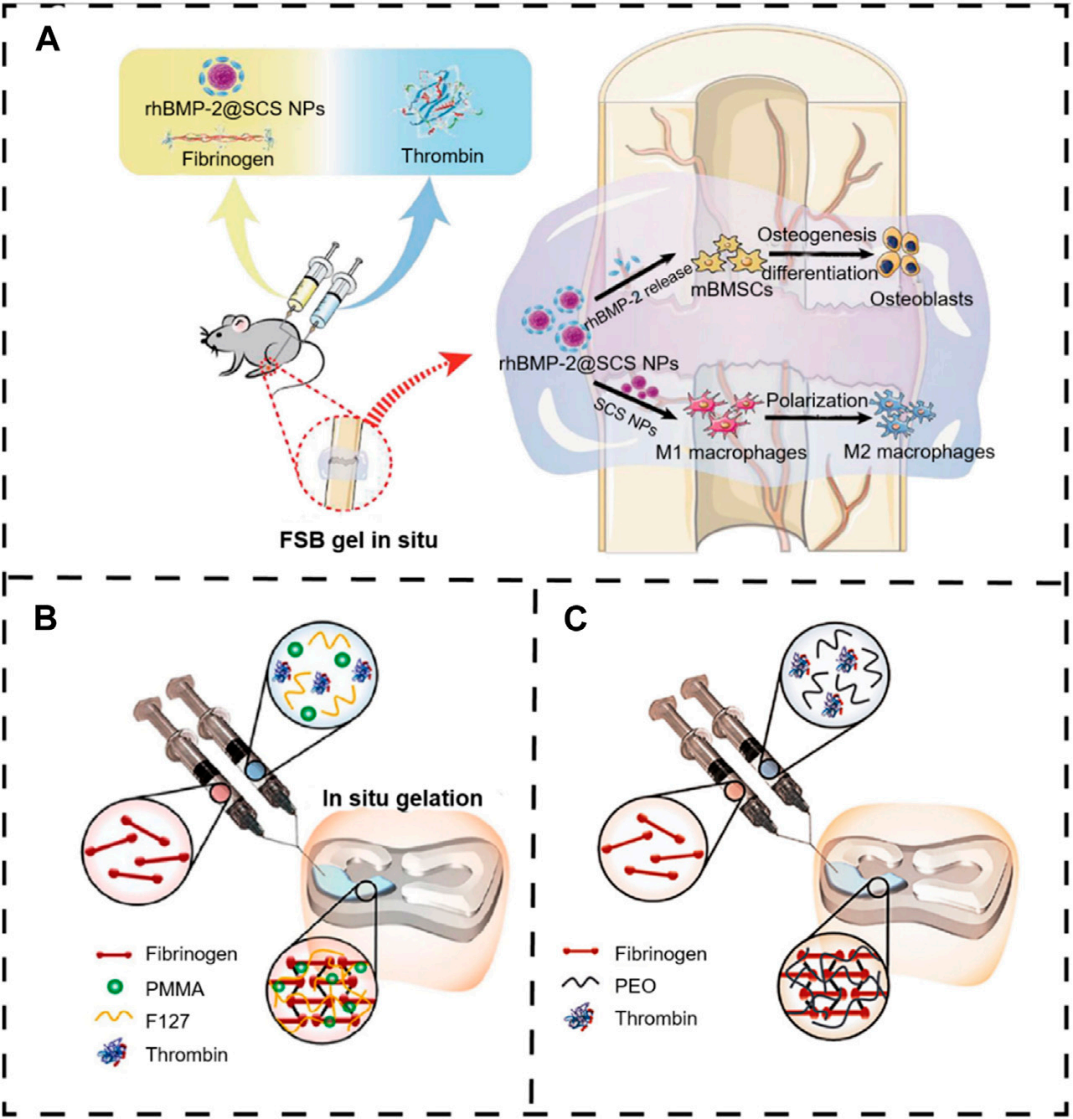
Fibrin-thrombin-based *in situ* hydrogels were applied in bone tissue disease. **(A)** Schematic illustration of rhBMP-2@SCS NPs to induce therapeutic osteogenesis (Chen et al., 2022). Copyright 2022, Elsevier. **(B)** Scheme of the gelation process of Fb/F127/PMMA hydrogel through a dual-syringe device while filling the meniscal defect region (An et al., 2021). Copyright 2021, SAGE Publications Ltd. **(C)** The schematic representation describes the overall procedure during the injection. The fibrinogen and thrombin (Tb) solutions were injected, and the semi-interpenetrated polymer network was subsequently formed (Kim et al., 2021). Copyright 2021, American Orthopaedic Society for Sports Medicine.

Thrombin, a serum protease found in the bloodstream, is specific to arginine-glycine bonds in fibrinogenic peptides. Thus, thrombin and fibrin are generally applied to tissue engineering as a field administration system (Noori et al., 2017). Injectable fibrin-based hydrogels have been extensively investigated for applications in bone tissue regeneration due to their minimally invasive implant procedure and shortened healing time, which reduces patient discomfort and complications and decreases healthcare costs (Kim et al., 2014). Furthermore, they can easily bridge faults of irregular shapes and provide the necessary mechanical support (van der Stok et al., 2015). During the final step of the biological polymerization process, soluble fibrinogen is cleaved by thrombin to produce insoluble fibrin, which binds to form fibers and a network. Meanwhile, a large number of red blood cells, platelets, and other entities are encapsulated in the network into a firm hydrogel system (Hickman et al., 2018). The *in situ* injection strategy offers the benefits of simple surgical procedures and the prevention of patient discomfort. Non-surgical strategies involving cellular or *in situ* hydrogels have been reported (Lyu et al., 2020). For example, Chen et al. (2022) developed a double-function injectable fibrin hydrogel with semi-synthetic chitosan sulfate nanoparticles that dramatically improves the level of recombinant human bone morphogenetic protein-2 (BMP-2) to induce osteogenesis and reduces the inflammatory response at the damage site. This fibrin-based hydrogel system effectively manages the release of BMP-2 and induces osteogenetic differentiation. Furthermore, the hydrogel system regulates the polarization of macrophages from the M_1_ to M_2_ stage with a significant decrease in inflammatory cytokines (Figure 11A). However, most injectables were composed of biomaterials that have not yet been clinically approved. In addition, they were also subjected to low mechanical stresses to reduce the load-bearing stresses applied to the meniscus. Therefore, mechanically stable, highly bioactive biomaterials must be developed for translational research using biomaterials approved by the FDA. For example, An et al. (2021) developed a clinically applicable and injectable semi-interpenetrated network (semi-IPN) hydrogel system based on fibrin, reinforced with F127 and polymethyl methacrylate (PMMA) to improve the intrinsic weak mechanical properties. Through the dual-syringe device system, the hydrogel could form a gel state within approximately 50 s, and the increment of compressive modulus of fibrin hydrogels was achieved by adding F127 from 3.0% (72.0 ± 4.3 kPa) to 10.0% (156.0 ± 9.8 kPa) (Figure 11B). Similar work has been reported by Kim et al. (2021), who fabricated a semi-IPN hydrogel system consisting of fibrin and polyethylene oxide (PEO) to improve the treating a segmental defect of the meniscus in a rabbit meniscal defect model (Figure 11C).

#### 4.2.4 Glucose oxidase (GOD)

**FIGURE 12:**
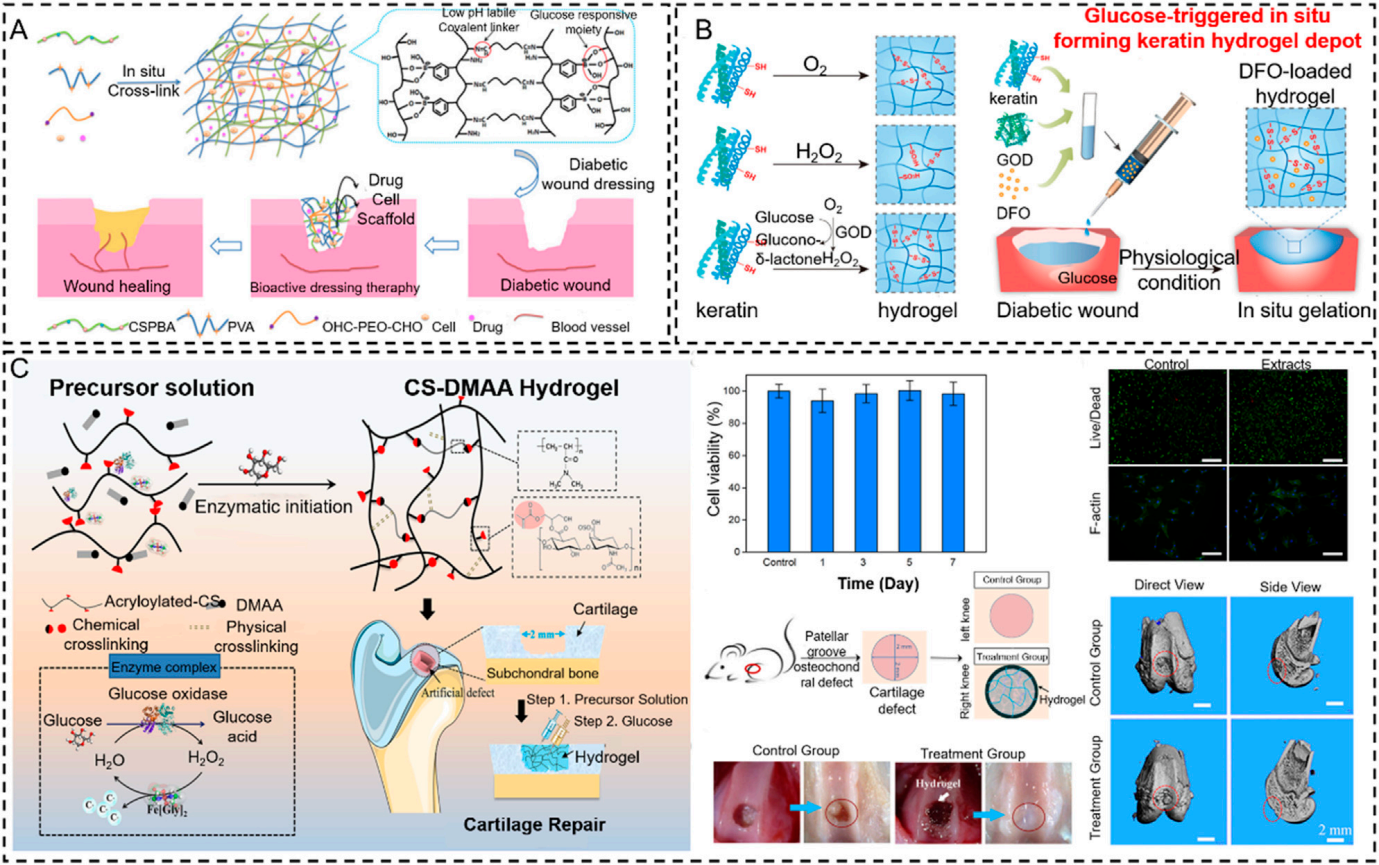
Glucose oxidase-based *in situ* hydrogels were applied in bone tissue engineering. **(A)** Scheme of pH and glucose dual-responsive injectable hydrogels with insulin and fibroblasts as bioactive dressings for diabetic wound healing (Zhao et al., 2017). Copyright 2017, American Chemical Society. **(B)** Schematic illustrations of gelation mechanisms by O_2_ oxidation, direct addition of H_2_O_2_ solution, and the indirect H_2_O_2_ supplement method via GOD-catalyzed glucose oxidation and glucose-triggered *in situ* forming keratin hydrogel as a drug depot for the treatment of diabetic wounds (Chen Y. et al., 2021). Copyright 2021, Elsevier. **(C)** Fabrication of the CS-DMAA hydrogel. The molecular structures of the acryloylated-CS and the poly (DMAA) are given. This hydrogel is used as a tissue filler via *in situ* injection and glucose-responsive hydrogenation (Zhang Q. et al., 2021). Copyright 2021, Wiley-VCH.

Glucose oxidase (GOD) is an important industrial enzyme in the food industry, textile bleaching and dyeing, biofuels, glucose biosensors, and other emerging fields. It is widely distributed in animals, plants, and microorganisms. Importantly, GOD can specifically catalyze the β-D-glucose to form gluconic acid and hydrogen peroxide under aerobic conditions. It offers the possibility of inducing *in situ* hydrogels by the cascade reaction from oxidation products. However, it has been applied to a special pathological model (diabetes) to achieve *in situ* hydrogel because it requires much glucose in this *in vivo* process. Thus, several works focus on GOD to induce an *in situ* hydrogel to treat wounds and bone tissue damage in patients with diabetes (Figures 12A,B) (Zhao et al., 2017; Chen Y. et al., 2021). For example, Zhang Q. et al. (2021) proposed a cascading enzyme polymerization process triggered by tissue fluids catalyzed by glucose oxide and ferrous glycinate, which improves the ultra-fast gelification rate of acryloylate and acrylamide chondroitin sulfates. The highly efficient production of carbon radicals and macromolecules helps the gel to rapidly polymerize, promoting soft tissue growth in bone defects. In addition, this copolymer hydrogel demonstrated potential for cartilage regeneration *in vivo* and *in vitro*. As a first example of using artificial enzyme complexes for *in situ* polymerization, this work provides a biomimetic approach to the design of force-adjustable hydrogels for bio-implantation and bio-printing applications (Figure 12C).

An enzyme is an active substance with high catalytic efficiency and specificity. Because of this, enzyme-catalyzed *in situ* hydrogels have a singular nature. Human enzyme catalysis is affected by the concentration of enzymes in the body. In addition, it is easy to lose enzyme activity when specific enzymes are introduced *in vitro* due to the special storage conditions of enzymes. These shortcomings limit the clinical application of enzyme-catalyzed *in situ* gels. In the future, vigorously developing synthetic nanozymes to replace natural enzymes may become a novel strategy to promote the development of enzyme-catalyzed *in situ* hydrogels.

### 4.3 Chemical reaction crosslinking stimulus-responsive *in situ* hydrogels

Compared with physical crosslinking, the *in situ* hydrogels produced by chemical crosslinking possess higher stability in the physiological state and showed excellent mechanical properties, gel stiffness, and slower degradation rates due to the covalent linking of the interpolymer chain (Chai et al., 2024). In addition, they exhibit flexible biodegradation and mechanical properties by adjusting hydrogel precursors and providing longer-lasting spatial support for slow regeneration processes (Pranantyo et al., 2024). Usually, those chemical reactions promote covalent linkage in hydrogels through chemical crosslinking, such as click chemistry (e.g., Diels–Alder reaction, Michael addition reaction, or thiol–Michael addition reaction). In the assembly of hydrophilic polymers, those deterministic groups, such as COOH-, OH-, and NH_2_-, are utilized to construct a hydrogel system via a covalent bond between amine-carboxylate and a Schiff alkali or isocyanate-OH/NH_2_ (Liu H. et al., 2023; Wang X. et al., 2024; Qu et al., 2024).

#### 4.3.1 Click chemistry

Recently, the development of click chemistry has presented a possibility to further enhance the specificity of *in situ* hydrogel systems. Click reactions are fast, spontaneous, versatile, and highly selective chemical conjugation reactions that can be generated under mild reaction conditions when two molecular substances or components are mixed or reacted together (Albada et al., 2021). Examples include strain-promoted azide-alkyne cycloaddition (SPAAC) (Liu et al., 2021) and inverse electron-demand Diels–Alder reactions, etc. (Vu et al., 2022). There are no potentially toxic catalysts in the progress of hydrogel crosslinking. Thus, click chemistry has expanded the research fields of tissue engineering and regenerative medicine (Jewett and Bertozzi, 2010).

**FIGURE 13:**
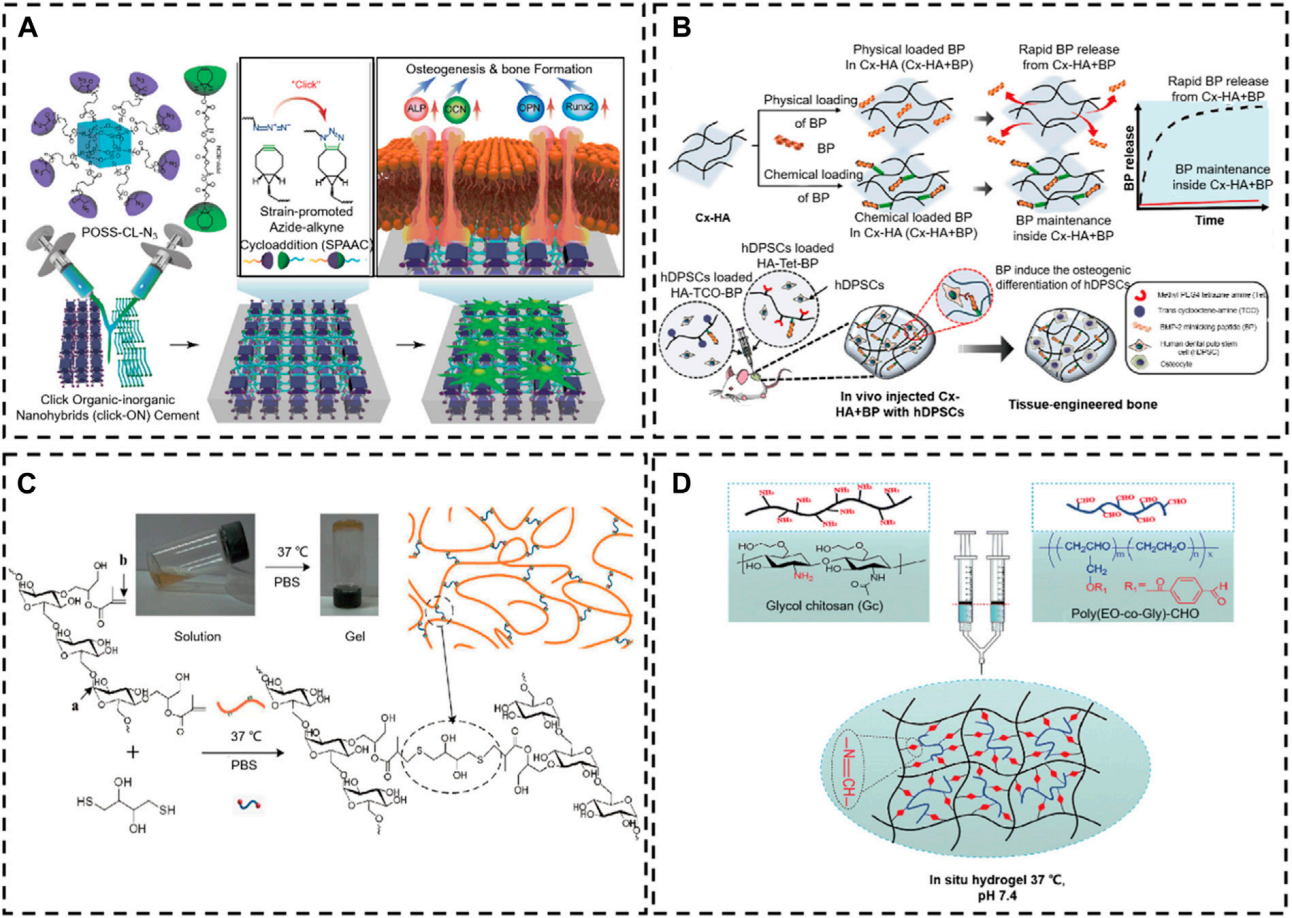
Chemical reaction crosslinking stimulus-responsive *in situ* hydrogel. **(A)** Oligomeric polyhedral oligomeric alkyne-azide cycloaddition (SPAAC) system (Liu et al., 2021). Copyright 2021, Elsevier. **(B)** Schematic illustration of the *in situ* hydrogel formed by a Diels–Alder click-crosslinking reaction. (Park et al., 2020). Copyright 2020, Elsevier. **(C)** Thiol-Michael addition reaction (Gilchrist et al., 2021). Copyright 2021, Elsevier. **(D)** Schematic representation of the formation of injectable hydrogels chemically cross-linked by a Schiff’s base reaction between aqueous solutions of GC and poly(EO-co-Gly)-CHO (Cao et al., 2015). Copyright 2015, RSC.

Due to the mild, safe, and effective reaction conditions, click chemistry offers the possibility of applying artificial chemical reactions *in vivo*. The wide application of click-through responses *in vivo* to increase the link between nanoparticles and target cells for drug administration has paved a new path in bone tissue engineering (Vu et al., 2022). For example, to obtain a quick gelling, Hermann et al. (2014) prepared a multi-arm PEG polymer modified by azide (a linear crosshair PEG-DBCO) via a SPAAC reaction between the azides and the BOD to apply in bone regeneration. Both polymers could be crosslinked in less than 30 s at ambient temperature and form a stable hydrogel structure. The hydrogel was developed to inhibit craniosynostosis via the release of the rmGremlin1 BMP inhibitor. After injection into the calvarial defect in a mouse model, this rmGremlin1 hydrogel successfully delayed synostosis in micro-CT images, demonstrating the potential of the SPAAC-based hydrogel. Similar work has been reported by Liu et al. (2021), who reported a new oligomeric polyhedral oligomeric SPAAC system of organic-inorganic nanohybrids (click-ON) that can be crosslinked without toxic initiators or catalysts. Click-ON scaffolding can also support high adhesion, proliferation, and osteogenesis of stem cells. *In vivo* evaluation results revealed exceptional bone formation with minimum cytotoxicity via a rat head malformation model. A high expression of alkaline osteogenic phosphatase and vascular marker CD31 was found at the defect site, which indicates an excellent regeneration capability for *in vivo* osteogenesis and vascularization (Figure 13A).

In addition, countless biomaterials (e.g., collagen, HA, alginate, dextran, chitosan, gelatin, silk, etc.) are modified by chemical bonds to achieve *in situ* click chemical crosslinking in physical conditions (Lao et al., 2023; Morrison and Gramlich, 2023; Rizwan et al., 2023; Tournier et al., 2023). For example, to prolong the degradation time of HA *in vivo*, Park et al. (2020). exploited tetrazine-modified HA and trans-cyclooctene-modified HA monomer to prepare an injectable *in situ* hydrogel system by Diels–Alder click-crosslinking reaction, which loaded a BMP2 mimetic peptide for improving bone tissue regeneration. After the *in situ* construction of hydrogel *in vivo*, this hydrogel system provided an excellent mimic ECM microenvironment for the human dental pulp stem cells (hDPSCs). The bone morphogenetic protein-2 (BMP-2) mimetic peptide is similar to BMP-2, which possesses an excellent ability to induce the osteogenic differentiation of hDPSCs. This HA-based *in situ* hydrogel quickly achieved sol-gel and formed a scaffold *in vivo*, which retained the BMP-2 for over a month. Thus, the HA-based *in situ* hydrogel is advantageous in inducing osteogenic differentiation of loaded hDPSCs (Figure 13B).

Furthermore, smart hydrogel systems, such as those triggered by UV (Sun S. et al., 2018; Jansen et al., 2018), NIR (Anugrah et al., 2019), and enzymes (Nguyen et al., 2019; Criado-Gonzalez et al., 2020), have been developed to induce the occurrence of click chemistry. For example, Ding et al. (2021) constructed a pH-responsive UV-cross-linkable chitosan hydrogel for actively modulating drug release behavior. The C_6_-OH selectively modified chitosan via a protection/deprotection strategy for the amino groups. In addition, the allyl groups on the C_6_ site and the amino groups on the C_2_ site endowed chitosan with UV crosslinking capability and pH responsiveness, respectively. A rapid gelling by UV crosslinking (30 s) with low-dose UV irradiation (4 mW/cm^2^) by thiol-ene was demonstrated for *in situ in vivo* microgels and hydrogels.

#### 4.3.2 Thiol-Michael addition reaction

The Michael addition reactions have been broadly applied in organic synthesis, such as material applications, surface modifications, polymer modifications, and polymer synthesis (Mather et al., 2006; Nair et al., 2014). Herein, we focus on a specific Michael addition reaction: the thiol-Michael addition reaction, which occurs between the thiol and vinyl groups and can be initiated by various catalysts, such as bases, metals, organometallics, and nucleophiles (Xiao et al., 2022). Among those catalysts, bases and nucleophiles are the most effective, with the least propensity to secondary reactions. In the traditional method of a thiol-Michael addition catalyzed by a base, the base extracts a thiol proton to produce a thiolate anion, which subsequently undergoes the addition of a Michael thiol. In the nucleophilic-mediated pathway, the nucleophile reacts with the electron-deficient double bond to produce an intermediate carbanion, which, in turn, deprotonates the thiol to produce a thiolate anion. The versatility provided by the weak sulfuric-hydrogen bond enables the thiol-Michael addition reaction to be initiated using a wide range of precursor materials.

The thiol-Michael addition reaction can be carried out under physiological conditions, which results in irreversible reactions in the bioengineering toolkit to fabricate a 3D hydrogel scaffold (Yu and Chau, 2012). For example, Gilchrist et al. (2021) described a functional gelatin hydrogel with maleimide, which can be crosslinked by a thiol-maleimide addition reaction for encapsulation of susceptible stem cell populations. This hydrogel system has tunable mechanics to match the bone marrow microenvironment for culturing hematopoietic stem cells *in vivo* without generating free radicals (Figure 13C). Liu et al. (2015) created a novel thiol-Michael addition reaction for *in situ* hydrogels that crosslinks dextran derived from glycidyl methacrylate and dithiothreitol. The encapsulation capacity of 3D cells is demonstrated by encapsulation of rat mesenchymal BSCs and NIH/3T3 fibroblasts in *in situ* hydrogels. The encapsulated cells maintain high cell viability.

#### 4.3.3 Schiff base reaction

Schiff base reactions are extensively applied to synthesize *in situ* hydrogels for bone tissue regeneration applications due to the mild reaction conditions and high reaction rate (Amirian et al., 2021). The Schiff reaction takes place between the amino (R-NH_2_) or hydrazide (R-NHNH_2_) and aldehyde (R-CHO) functional groups of imine or acrylic hydrazone. It has grown into one of the most popular strategies for developing biocompatible hydrogels in tissue engineering (Wang B. et al., 2021; Hu et al., 2021). Recently, various biomaterials have been explored for utilization in Schiff base *in situ* hydrogels (Lee, 2018; Hernández-González et al., 2021). Chitosan is an excellent biomaterial for the preparation of *in situ* hydrogel systems based on Schiff reactions because of the abundance of amino groups on its spinal column (Li Z. et al., 2021). For example, Cheng et al. (2014) reported *in situ* chitosan polysaccharide hydrogel for both cellular and protein administration. The chitosan polysaccharide is crosslinked via an imine bond resulting from the Schiff base reaction between the amino functionalities of chitosan and the aldehyde groups of the dextran aldehyde precursors. This approach eliminated the potential cytotoxicity and improved the biocompatibility of polymers without additional crosslinking agents. In addition, this work demonstrated the versatility of the gel in terms of manufacturing and the ability to change the mechanical properties by adjusting the extent of crosslinking. Cao et al. (2015) successfully explored a functionalized multi-benzaldehyde analog PEG, poly (ethylene-co-glycidol ether)-CHO (poly (EO-co-Gly)-CHO), and glycol chitosan to develop an *in situ* hydrogel system for cartilage tissue repair. This multi-functionalized hydrogel system has been chemically crosslinked by a basic Schiff reaction between the chitosan glycol amino groups and polyaldehydes (EO-co-Gly)-CHO groups under physiological conditions (Figure 13D). The results indicated that the *in situ* hydrogel system not only possesses excellent adjustable physical properties (e.g., water uptake, degradation, mechanical properties, and network morphology) but also has the excellent ability to induce chondrogenesis. Furthermore, other *in situ* hydrogels based on biomaterials coupled with the crosslinking of the Schiff base have been extensively studied in tissue engineering (Antony et al., 2019; Chen J. et al., 2021).

Chemical reaction crosslinking stimulus-responsive *in situ* hydrogels were formed by special functional groups between molecules in precise crosslinking and stable. However, the functional groups or reaction products of these chemical grafts can potentially be toxic. This will be detrimental to the application and registration of gel products. Therefore, it is necessary to improve and optimize the safe chemical grafting preparation process in order to promote the industrialization development of the chemical reaction crosslinking stimulus-responsive *in situ* hydrogels.

### 4.4 Ion crosslinking stimulus-responsive *in situ* hydrogel

Metal ions are an indispensable microelement in regulating whole-body homeostasis (Zhang et al., 2024b). They can be elaborately designed by linking with polymer chains to transform *in situ* functional hydrogels to improve bone tissue regeneration (Zhang P. et al., 2024). Ions can recognize biomaterial monomers, promote crosslinking between molecules, and enhance the stability of the structure. Therefore, the ion-triggered biomaterials that form *in situ* hydrogels have received extensive research attention (Ramírez et al., 2024). Alginate, a naturally derived polysaccharide, has been regarded as an ideal hydrogel biomaterial for bone tissue engineering because of its biocompatibility, tunable mechanism properties, low immunogenicity, and ease of gelation by metal ions (Li et al., 2024). It is generally believed that this crosslinking mechanism is the interaction of two carboxyl groups on the adjacent polymer chain with divalent cations to form an ion bridge or chelate with ions via the hydroxyl and carboxyl groups on polymer chains. Thus, alginate-based hydrogel systems can be flexibly manufactured to achieve metal ions response (e.g., Ca^2+^, Si^2+^, etc.) (Chen et al., 2024; Li et al., 2024; Zhong et al., 2024). Usually, alginate-based hydrogels are prepared by contacting alginate precursors with CaCl_2_ aqueous solutions. However, the gelling rate is too fast and difficult to control when using CaCl_2_ as a crosslinker. Thus, various crosslinking retardation agents have been applied to tune the gelation rate to form an injectable *in situ* alginate-based hydrogel (Nandi et al., 2008). For instance, Jung et al. (2018) prepared *in situ* gel alginate (ALG)/HA hydrogel system with a controlled gelatin rate by blending CaSO_4_ and Na_2_HPO_4_ as crosslinking retardation agents. The ALG/HA hydrogels possess a controlled gelation rate *in vivo*. It is possible to stabilize and control the release of bioactive molecules (BMP-2 immobilized in the hydrogel for 5 weeks) for bone regeneration. The results of cell culture and animal studies on micropigs revealed that osteogenesis differentiation of hBMSCs improved with the increase of HA components in BMP-2-stimulated ALG hydrogels. In addition, hBMSCs/BMP-2 loaded in ALG/HA hydrogels can extensively enhance bone regeneration by synergistic action compared to single ALG/HA hydrogels.

Metal-chelating hydrogels are physical hydrogels that are completely crosslinked by complexes between ligand-modified polymers and metal ions. The mechanical properties of these hydrogels depend on the density and kinetics of the metal coordination interaction (Raymond et al., 2015). For example, HA and the catechol compound gallic acid (GA) have iron coordination activity. The conjugates of HA and GA (HA-GA) immediately form the hydrogel in the presence of the Fe^3+^ ion. After subcutaneous injection into mice, HA-GA and Fe^3+^ ions form an *in situ* hydrogel and remain at the injection site for at least 8 days (Ko et al., 2019). The ions in the ion crosslinking can replenish the trace elements of the human body. However, the amount of ions in the gel is much higher than the normal level of ions in the human body. Therefore, the safety of the tissues surrounding the gel must be thoroughly evaluated and studied.

## 5 Conclusion and outlook

Stimuli-responsive *in situ* hydrogel delivery platforms have demonstrated great potential in repairing various kinds of bone damage (e.g., accidents, cancer, or age). Recent years have witnessed rapid progress in the development of *in situ* hydrogel systems based on different functional biomaterials and stimulus-response approaches to treat bone-related diseases. This review summarized the various strategies of the smart responsive *in situ* hydrogel systems in bone tissue engineering. The two strategies of exogenous and endogenous stimulus response are detailed and introduced. The exogenous stimulus-responsive *in situ* hydrogel systems usually require exogenous trigger apparatus, such as ultraviolet light, near-infrared light, and ultrasonic apparatus, to form gels. In this process, those nature or synthesis biomaterials are usually modified by functional groups (e.g., unsaturated bonds) or combined with functional nanoparticles (e.g., MoS_2_, Cu_2_O, F_3_O_4_, etc.) to match special trigger approaches. However, the endogenous stimulus-responsive *in situ* hydrogel systems, which are free of exogenous trigger apparatuses, relay on the physical microenvironment (e.g., temperature, enzyme) or chemical reactions (click chemistry, thiol-Michael addition reaction, Schiff base reaction) to form gels *in vivo*. Moreover, we analyzed the research status and advantages and disadvantages of different kinds of stimulus-responsive *in situ* hydrogels. It appears that temperature-sensitive hydrogels will be most promising for clinical application. The specific reasons are: 1) Temperature-sensitive hydrogels can form *in situ* hydrogels at human body temperature without the need for manipulation and specific functional modifications. 2) The simple composition of temperature-sensitive hydrogels both reduces cytotoxicity and opens the possibility for industrial production. 3) Temperature-sensitive hydrogels only need to be injected once to achieve gelatinization therapy *in vivo*, which can greatly improve patient compliance and is more suitable for clinical applications.

Numerous studies have confirmed that smart responsive *in situ* hydrogel technology plays a significant role in bone tissue engineering, which provides a theoretical foundation for future clinical applications. Although excellent regeneration effects have been achieved, research is still in its infancy. Clinically, there are many limits to the development of a reliable, efficient, smart, responsive *in situ* hydrogel system. First, the systems are subjected to advanced trigger conditions, so it is necessary to develop novel stimulation methods. Second, the rapid rate of degradation of the hydrogel platform must be adjusted to be consistent with the rate of regeneration of the bone defects. In addition, the poor mechanical stability of the hydrogel system fails to match bone tissue, including rapid degradation and burst release, poor integration with native cells, low mechanical stability, and immunogenicity.

Despite the significant challenges, the development of smart responsive *in situ* hydrogel-based bone regeneration is extremely promising for the future treatment of bone diseases and damage. With a better understanding of hydrogels, bone abnormalities, ECM, and how they interact, hydrogels will undoubtedly become a powerful tool for the clinical treatment of bone abnormalities.
